# Environmental Impact of Digital Health Care Technologies in Reducing Carbon Footprints: Scoping Review

**DOI:** 10.2196/88341

**Published:** 2026-10-21

**Authors:** Sarath Rathnayake, Chinasa Odo, Natasha Alvarado, Hadiza Ismaila, Veronica Parisi, Joshua Pink, Ruth Agbakoba, Rebecca Randell

**Affiliations:** 1 Centre for Digital Innovations in Health and Social Care, Institute of Health and Social Care, University of Bradford, Bradford, England, BD7 1DP, United Kingdom, +44 1274236181; 2 Wolfson Centre for Applied Health Research, Bradford, United Kingdom

**Keywords:** carbon footprinting, digital technologies, environmental impact, health care, scoping review

## Abstract

**Background:**

Globally, the carbon footprint of the health care sector is significant and growing, and digital health care technologies (DHTs) offer unique opportunities to reduce the carbon footprint of health and care systems. However, their environmental impact has not been adequately investigated.

**Objective:**

This scoping review aims to explore the environmental impact of DHTs, focusing on reducing their carbon footprint and evaluating methods for assessing that impact.

**Methods:**

This scoping review followed the method outlined by Arksey and O’Malley, adhering to the PRISMA-ScR (Preferred Reporting Items for Systematic Reviews and Meta-Analyses extension for Scoping Reviews) guidelines. The databases of MEDLINE, Scopus, and Web of Science were searched for studies published in English with no time limitation. Search terms were categorized under three broader concepts: “Digital Health Technologies,” “Health and Care,” and “Carbon Footprint.” Studies were included based on screening titles and abstracts, followed by review of full-text papers by at least 2 reviewers. Narrative synthesis was used to report the findings.

**Results:**

The database search yielded 4274 studies. Following screening and reference list checks, 70 studies were selected for inclusion. The earliest included study was published in 2007, with the majority originating from the United States (n=26) and the United Kingdom (n=12). Most studies assessed telemedicine or telehealth interventions. Retrospective analyses predominated, although prospective, cross-sectional, mixed method, and life cycle assessment (LCA) designs were also reported. Environmental impact was most often evaluated through travel-related carbon savings, with some studies incorporating energy use, medical equipment or resources, infrastructure, or waste. LCA studies applied process-based approaches that included detailed activity- and product-specific data, as well as Environmentally Extended Input-Output (EEIO) models. In most cases, carbon footprint measurements were based on assumptions, with calculations relying on published emission factors or calculators. Some studies have expressed CO_₂_ emissions using emission equivalencies, such as equivalent carbon sequestration and equivalent CO_₂_ emissions. Overall, DHTs demonstrated positive environmental impacts by reducing carbon footprint. However, negative impacts, especially electricity consumption and the lifecycle impacts of digital devices, were also noted.

**Conclusions:**

DHTs are associated with reductions in carbon emissions and related environmental benefits, with telemedicine showing potential to lower the carbon footprint of the health care sector. However, evidence highlights the negative impacts associated with energy demand and the full lifecycle of digital devices. Given the substantial heterogeneity in assessment methods and outcome measures across studies, these findings should be interpreted with caution. To capture the true environmental implications of digital health, more rigorous, standardized, and comprehensive assessment methods are required. Bridging these methodological gaps is essential to ensure that DHTs not only support sustainable health care delivery but also make a meaningful contribution to mitigating global climate change.

## Introduction

Climate change poses a serious threat to human health, exacerbating disease burdens, food insecurity, and mortality related to extreme weather [1]. The rise in greenhouse gases (GHGs), such as carbon dioxide (CO_2_), methane (CH_4_), and nitrous oxide (N_2_O), is a major contributor to global warming and climate change [2]. Increased CO_2_ emissions and their consequences, such as global warming and extreme weather events, have highlighted the need to reduce carbon footprints. Carbon footprint refers to the total GHG emissions, expressed as CO_2_ equivalents (CO_2_e) [3]. Wiedmann and Minx [4] define carbon footprint as “a measure of the exclusive total amount of carbon dioxide emissions that is directly and indirectly caused by an activity or is accumulated over the life stages of a product.”. The term “product” includes both goods and services [4]. In broader terms, the carbon footprint encompasses emissions arising from the activities of individuals, populations, organizations, governments, companies, processes, and industry sectors [4]. Reducing these emissions has become a global concern [5]. The health care sector is a major contributor to global GHG emissions, accounting for approximately 4.6% [6], driven by energy use, transportation, and the life cycle of products, including their manufacture, use, and disposal [7]. The emissions include direct emissions from health care facilities and health care–owned vehicles (17%), indirect emissions from purchased energy sources such as electricity, steam, cooling, and heating (12%), and the largest proportion of emissions derived from the health care supply chain (71%) [7]. The United States, China, and the European Union account for more than half of the health-related global carbon footprint [7]. Despite the significant size of the health care sector, it has only recently begun to recognize and address this issue [6].

Health care is constantly evolving with the integration of new digital interventions into everyday practice [8]. Digital health care technologies (DHTs) have become an integral part of health care, with the potential to offer benefits that are ethical, safe, secure, reliable, equitable, and sustainable [9]. DHTs include the digitalization of various facets of the health and care system [10], including telehealth, electronic health records (EHRs), wearable devices, mobile health (mHealth) applications, Internet of Things (IoT), and AI. Recent systematic review evidence suggests that digital health interventions can enhance health care accessibility, support chronic disease management, improve patient adherence, and increase clinical efficiency, while also contributing to reductions in carbon emissions, health care–related energy use, and medical waste [11]. Among these technologies, telemedicine is the most widely used approach, involving the use of electronic communication technologies such as video conferencing, email, text messaging, patient portals, and remote monitoring to facilitate communication between patients and health care providers, thereby supporting and enhancing patient care [12]. DHTs offer a range of potential advantages for health care, including improving access to care, empowering patients, enhancing efficiency, improving the quality of health care and outcomes, enabling data-driven decision-making, enhancing collaboration, and reducing costs [8,13]. Moreover, these technologies have promising effects on environmental impact, particularly the reduction of GHGs [5,14] by minimizing the need for physical infrastructure, reducing travel for in-person consultations, and optimizing resource use [6]. For example, recent systematic reviews demonstrated that telemedicine interventions help reduce travel-related CO_2_ emissions by reducing the need for in-person consultations and care [15-18].

Today, researchers assess the environmental impact of DHTs to understand and reduce their carbon footprint and resource use, and to improve the environmental sustainability of clinical care while supporting sustainable health care innovation [19,20]. A recent review has highlighted that the environmental impacts of health care digitalization are complex, showing both potential reductions and unintended increases in carbon emissions depending on system design, infrastructure demand, and patterns of use [21]. Life cycle assessment (LCA) is widely accepted as a comprehensive assessment that evaluates environmental burdens and impacts across the sequential and interconnected stages of a product’s life cycle, from the extraction or generation of raw materials through manufacturing and use to final disposal [22]. Recent evidence reported that carbon footprint analyses often lacked transparency, employed differing system boundaries, and used nonstandardized environmental metrics, limiting the comparability of findings and the ability to determine the overall climate impact of health care digitalization [11,22]. These findings highlight the need not only to understand the environmental impacts of DHTs but also to examine the methods used to assess and report those impacts across studies.

This scoping review aimed to explore the environmental impact of DHTs, focusing on reducing their carbon footprint and evaluating methods for assessing that impact. It provides a comprehensive understanding of the environmental benefits and challenges associated with DHTs by synthesizing evidence on both their carbon footprints and the methods used to assess them, thereby highlighting the strengths, limitations, and consistency of current evaluation approaches and informing future research, practice, and policy development.

## Methods

This review followed the scoping review method proposed by Arksey and O’Malley [23], consisting of 5 steps: identifying the research question, identifying relevant studies, selecting the studies, charting the information and data, and collating, summarizing, and reporting the results, providing a structured approach. Scoping reviews are increasingly popular for reviewing health research evidence and often involve mapping, which summarizes a range of evidence to convey the breadth and depth of a field [17-19]. We adhered to the PRISMA-ScR (Preferred Reporting Items for Systematic Reviews and Meta-Analyses Extension for Scoping Reviews) guidelines [24] when developing the search strategy and reporting the findings.

### Review Questions

This scoping review addressed the following research questions:

1. What types of DHTs are currently implemented to reduce carbon footprints in health care settings?
2. What data collection methods are employed to assess the environmental impact of DHTs within health care settings?
3. What strengths and limitations and/or gaps exist in the data collection methods for measuring the environmental impact of DHTs?
4. What is the environmental impact of DHTs currently available in different health care settings?

### Search Strategy

The search strategy was developed with the help of an information specialist (VP), who tested the initial search and reviewed literature to identify relevant terminology. The relevant studies were identified by searching MEDLINE via EBSCOhost, SCOPUS, and Web of Science. Search terms were categorized under 3 broader concepts Digital Health Technologies, Health and Care, and Carbon Footprint . Both keywords and MeSH terms were applied (Multimedia Appendix 1). Searches were run up to February 2025, and no date restrictions were applied to ensure that no relevant evidence was missed. Finally, the reference lists of all studies included following full-text review were examined to identify additional potentially relevant studies. The MEDLINE via EBSCOhost search strategy is detailed in Multimedia Appendix 2.

### Inclusion and Exclusion Criteria

We included primary studies, including qualitative, quantitative, and mixed method studies, that examined the environmental impact of DHTs, regardless of the care setting or geographical location, and were published in English. Studies that addressed only nonenvironmental outcomes, such as clinical efficacy or economic evaluation, or that were conducted in non–health care settings were excluded. Additionally, review papers, opinion pieces, editorials, and conference abstracts were excluded. We excluded review papers because this review aimed to map evidence from primary studies on the environmental impacts of DHTs and to examine the methods used to assess these impacts. Including review papers could have resulted in duplication of evidence. As our database search was not restricted by publication date and covered the period from inception to the search date, we anticipated that most relevant primary studies would be identified and included in our review.

### Screening and Study Selection

Sources identified through database searches were imported into Covidence, and duplicates were automatically removed. Six reviewers (SR, CO, NA, HI, JP, and RA) selected the studies through a 2-phase screening process: (1) titles and abstracts screening and (2) full-text screening. Titles and abstracts, and then full-text studies, were independently screened by at least 2 reviewers based on predefined inclusion and exclusion criteria. Conflicts were resolved through periodic consensus meetings.

### Quality Appraisal

This scoping review aimed to map the existing literature on the environmental impact of DHTs in health and social care. Most included studies were retrospective studies or record or secondary analyses. Consistent with established guidelines for scoping reviews, a formal quality appraisal was not undertaken [25]. Because the primary objective of this scoping review was to identify, describe, and synthesize the available evidence rather than to evaluate the effectiveness of interventions, and study selection was based solely on the relevance of information. However, we extracted the study design, along with reported strengths and limitations of environmental assessment methods and techniques, to provide context for interpreting the findings.

### Data Extraction

The data extraction template was developed in Covidence based on input from the review team. Three reviewers (SR, NA, and CO) piloted this data extraction template by extracting data from 3 full-text papers and necessary changes were made. It comprises general study information (author names, year published, location or country, care setting, type of study design, type of DHT, and participants), environmental impact assessment approaches, GHG emission assessment (method used, findings, strengths and limitations of assessment methods, and recommendations), carbon footprint assessment (methods used, findings, reliability, validity, and strengths and limitations of assessment methods) and overall conclusion related to environmental impact. The first author (SR) completed the data extraction. To enhance accuracy and consistency, a subset of the extracted data (14/70, 20%) was verified by a second reviewer (JP).

### Data Analysis, Synthesis, and Presentation

A narrative synthesis was used to analyze and report the findings by categorizing key concepts and summarizing them thematically. The findings were then organized into 5 main sections. The first section includes a summary of the selected studies, covering the author, year of publication, and country; the type of digital technology and any comparator; the study setting, design, and participants; and the environmental impact assessment methods. Section 2 includes the types of DHTs assessed for environmental impact. Section 3 includes environmental impact assessment methods and techniques. Section 4 includes the strengths and limitations of environmental impact assessment methods and techniques. Section 5 includes the environmental impact of DHTs.

## Results

### Search Outcomes

A total of 4274 papers (1761 from Scopus, 1313 from MEDLINE, and 1200 from Web of Science) were identified through database searches. After removing duplicates (n=1978), 2296 titles and abstracts were screened based on inclusion and exclusion criteria. We excluded 2159 records and selected 137 sources for full-text review. Three full-text papers could not be retrieved. Therefore, 134 full-text papers were screened, and 64 papers were included for data extraction. Multimedia Appendix 3 lists the excluded studies. An additional 6 papers were identified through reference list screening of the included studies. Finally, we included 70 studies [12,26-94] in the final data extraction (Figure 1).

**Figure 1.**
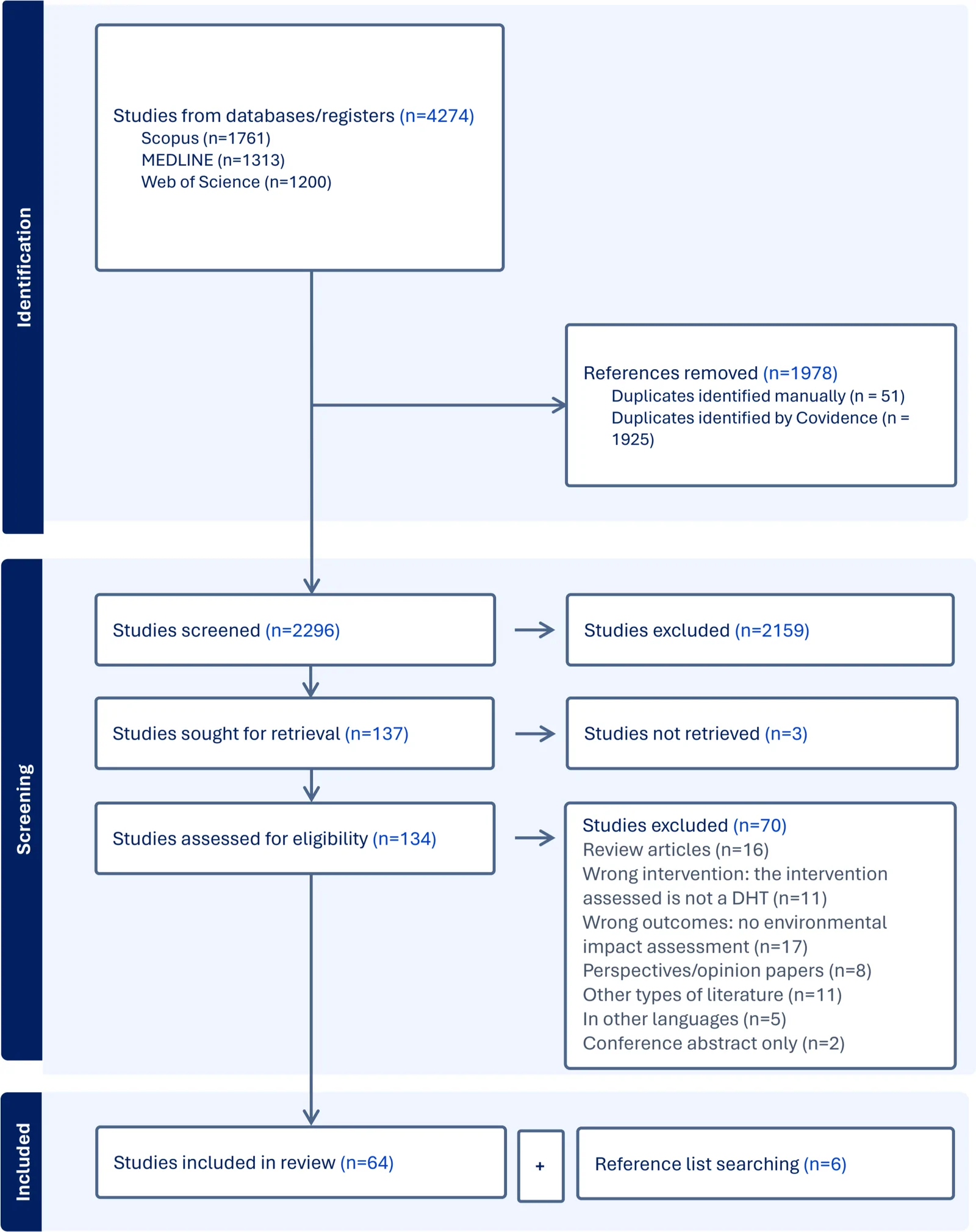
PRISMA flowchart: identification of studies from databases and reference list search. DHT: digital health care technology.

### Descriptive Summary of the Included Studies

A detailed summary of the included studies is presented in Multimedia Appendix 4. The earliest study was published in 2007. Most studies (n=57) were published after 2020, following the COVID-19 pandemic. The majority of studies were conducted in the United States (n=26) [26-51] and the United Kingdom (n=12) [52-63]. Other studies were from Canada (n=5) [64-68] and Australia (n=4) [69-72]; 3 studies were from Italy [73-75]; 2 studies were from Brazil [76,77], India [78,79], Spain [80,81], and Germany [82,83]; and one study from France [84], Netherlands [85], China [86], Turkey [87], Austria [88], Croatia [89], Serbia [12], Ireland [90], Switzerland [91], Denmark [92], Portugal [93], and Sweden [94] (Figure 2).

**Figure 2.**
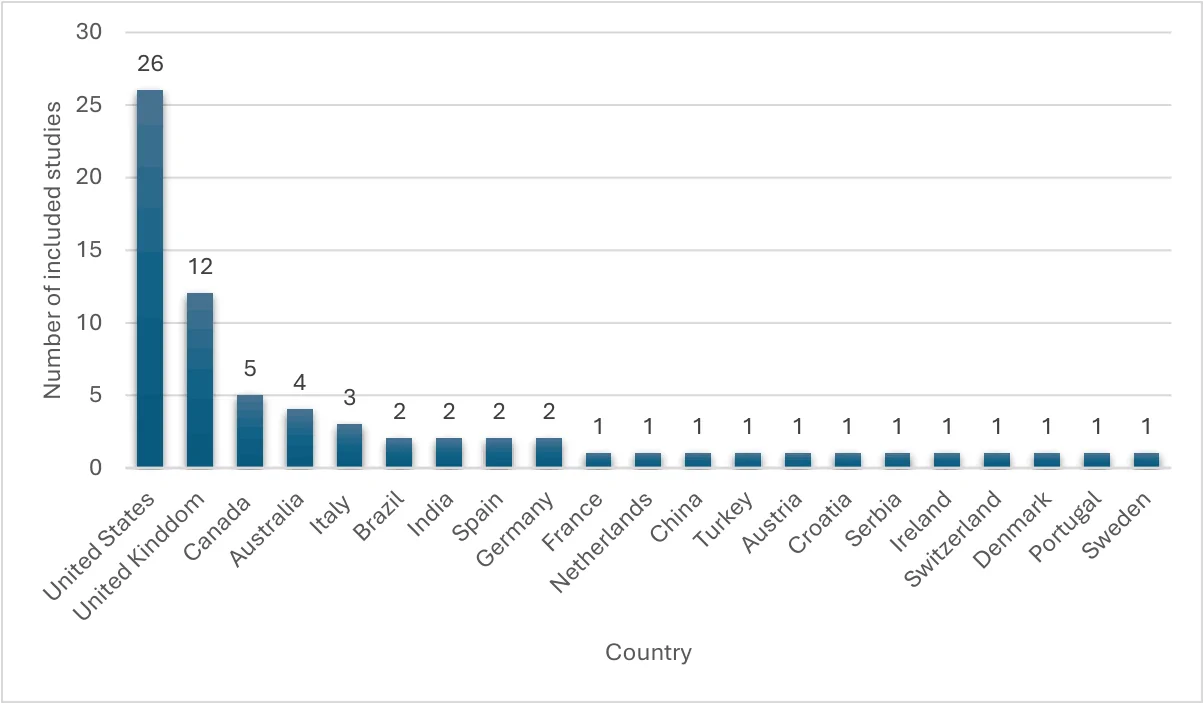
Geographic distribution of included studies.

The majority of studies were conducted in hospitals or hospital-based care units [12,27,35,38,40,45,47,50,52,53,57,59,62,65,67,69-75,79-81,86,92-94] and academic settings, including hospitals, health centers, departments, or clinics [29-34,37,41-43,48,64,66,76,77,82-84,87,88]. Some studies were conducted in primary and community care settings, for example, primary care centers [80,89,91], elderly living schemes [60], and community wound care centers [70]. Some studies were based on out-patient care settings, including care centers or mixed care facilities such as out-patient [36,54,55], cystic fibrosis centers [85], out-patient clinics [70], tertiary care centers or clinics [28,58]; tele-endoscopy remote clinics [56]; urology clinics [90], primary and tertiary eye care units [78], geriatric medicine clinic [61]; general surgery subspecialty clinic [39]; tertiary vascular center [63]; pediatric surgical care facility [44]; and cancer care unit [68]. Some studies were based on national and platform-based telehealth systems, such as the National Telehealth Platform [43], the Veterans Affairs (VA) health care system [46], Cancer Care Institute [44], and Kaiser Permanente Health System [26].

The most frequently used study design was retrospective analysis, including retrospective observational studies, retrospective cohort studies, and record analysis [12,26-31,33-39,42-45,47-50,52,54,55,61,62,64,68,69,74,77,79,80,84-87,90,91]. Several studies employed prospective designs, including prospective surveys and prospective quality studies [50,53,58,59,71,78] and cross-sectional studies, including surveys [46,57,66,76,83,93]. Other designs used were mixed method studies [40,70,72,92], secondary data analysis [65,89,94], cohort studies not identified as either retrospective or prospective [63,67,88], LCA [41,60,73], quality improvement studies [32,75], feasibility studies [56], and a randomized controlled trial [82] (Figure 3).

**Figure 3.**
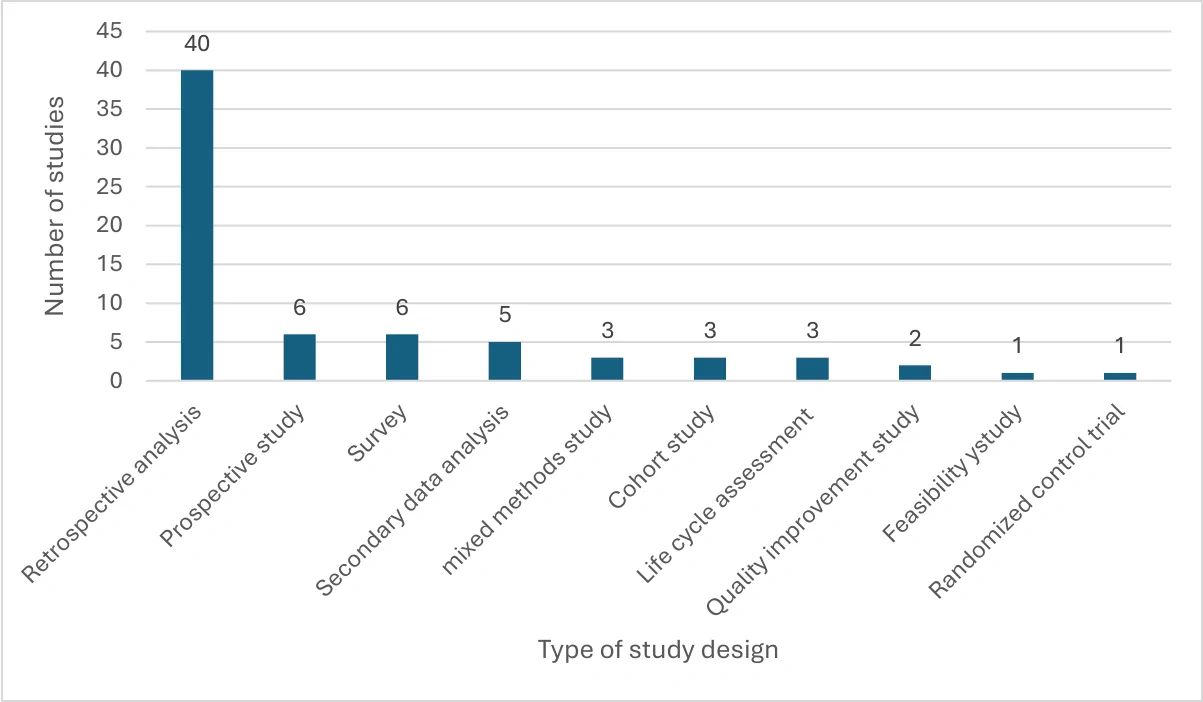
Type of study design.

### Type of DHTs Assessed for Environmental Impact

When assessing environmental impact, most studies (n=65) focused on virtual interventions or systems. Other digital health interventions included EHRs [26,79], eHealth applications [89], digital health and well-being services [60], and a digital health app combined with video consultations [82]. In virtual interventions, the focus was on telehealth and telemedicine, but different terms or aspects were often used to describe these telehealth interventions. Such interventions included virtual appointments, teleconsultations, telemedicine, or telehealth visits [12,28-33,36,37,43-54,56,57,59,61-69,71-76,80,81,83-88,90-93]; virtual clinics or remote teleclinic [38,41,55,58,62]; tele dentistry [27]; remote monitoring [85]; digital wound management platforms [64]; teleophthalmology [78], telehealth screening [34,35]; telerehabilitation [40,94]; and electronic consultation [42].

In telehealth and telemedicine, the leading technology used was video technology (online or audiovisual platforms) [12,29,31,38,40,47,49,50,57,59,64,68,69,72,73,76,78,83,84,86-88,92-94]. Some studies used telephone calls [34,35,53,55,58,71,74,90]. Some other studies used both video technology and telephone calls [28,33,36,41,44,46,48,52,54,61,62,65,66,85]. One study used audio-only and audio-video telehealth services [37]. Other technologies used included tele-endoscopy images with video conferencing [56], remote review through EHRs and telephone calls [80], Zoom (Zoom Communications, Inc) technology [27], eConsult services [42], and photography [63]. In studies on virtual technologies, many researchers used in-person visits or patients receiving usual care as a comparator [27,28,31,35,39-41,43,44,47,48,51-55,61,63,65-67,69,71,73,81-84,87,88,91-94]. In one study [79], paper documentation was used as a comparator to electronic documentation (Multimedia Appendix 4).

### Environmental Impact Assessment Methods Used

A variety of methods are used to assess the environmental impact of DHTs across different contexts, as presented in Table 1.

**Table 1.** Environmental impact assessment methods used.

Environmental impact assessment method	References
Assessment of travel distance saved	Smith et al [69] Materazzo et al [74]
Assessment of CO_2_^a^ emissions based on travel	Danesh et al [27] Kellerman et al [28] Wong et al [30] Mojdehbakhsh et al [32] Robinson et al [34] Thota et al [38] Iaccarino et al [40] Cockrell et al [44] Jiang et al [46] Sillcox et al [47] Penaskovic et al [48] Cummins et al [49] Patel et al [50] Grabski et al [51] Dorrian et al [56] Lewis et al [57] Galts et al [66] Lambert et al [68] Barakat-Johnson et al [70] Schulz et al [71] Papavero et al [75] Desenzi Ciaralo et al [76] Prosperi Desenzi Ciaralo et al [77] Rani et al [78] Arndt et al [83] Meryk et al [88] Croghan et al [90] Dieperink et al [92]
Assessment of GHG^b^ emissions	Dullet et al [31] Roy et al [33] Moore et al [42] Sharma et al [43] Gunn et al [45] Masino et al [64] Vidal-Alaball et al [80] Muschol et al [82]
Assessment of other environmental pollutants	Dullet et al [31] Gunn et al [45] Masino et al [64] Vidal-Alaball et al [80] Muschol et al [82]
Carbon footprint assessment	
LCA^c^ as a comprehensive method	Martinez et al [29] Sillcox et al [39] Tarpani and Gallego-Schmid [60] Savoldelli et al [73] Kwon et al [79] Giacomo P and Håkansson [89] Holmner et al [94]
LCA based on the	
Input-output model	Wang et al [35]
ISO 14040 standards	Thiel et al [41]
Comprehensive footprint approach	Schmitz-Grosz et al [91]
Hybrid life-cycle assessment approach addressing environmental impact from both bottom-up (prospective item process analysis) and top-down (using a national economic approach to input–output analysis) directions	Lathan et al [63]
Echo-health footprint model	Heffernan et al [67]
Carbon footprint assessment considering only one or a few aspects	
Travel	Marković et al [12] Roy et al [33] Wang et al [35] Dacones et al [36] Whetten et al [37] Moore et al [42] Jiang et al [46] Moore et al [52] Miah et al [53] King et al [54] Bhavanasi and Kulkarni [55] Connor et al [58] Sellars et al [59] Bartlett and Keir [61] Blenkinsop et al [62] Masino et al [64] Mainer-Pearson et al [65] Maria et al [81] Filfilan et al [84] Oppelaar et al [85] Özen et al [87] Oliveira et al [93]
Energy consumption	Bhavanasi and Kulkarni [55] Blenkinsop et al [62] Masino et al [64] Filfilan et al [84]
Use of infrastructure	King et al [54]
Telephone communication	Bhavanasi and Kulkarni [55] Bartlett and Keir [61]
Personal protective equipment	Bhavanasi and Kulkarni [55] Bartlett and Keir [61]
Water usage	Bhavanasi and Kulkarni [55] Bartlett and Keir [61]
Preoperative testing	Wang et al [35]
Waste	Bartlett and Keir [61]
Heating and lighting	Bartlett and Keir [61]
Use of CO_2_ emission equivalencies (communicating the environmental impact in a more relatable way)	
Equivalent carbon sequestration	
Carbon offsetting via trees	Iaccarino et al [40] Gunn et al [45] Patel et al [50] Lewis et al [57] Connor et al [58] Lathan et al [63] Galts et al [66] Barakat-Johnson et al [70] Oppelaar et al [85] Croghan et al [90]
Carbon sequestration equivalent-land in acres	Turley et al [26] Thiel et al [41] Patel et al [50]
Equivalent CO_2_ emission	
Passenger vehicle emissions	Penaskovic et al [48] Patel et al [50]
Home electricity usage	Iaccarino et al [40] Thiel et al [41] Patel et al [50]
Home energy use	Patel et al [50]
Amount of gasoline consumed	Cockrell et al [44] Patel et al [50] Galts et al [66]
Charging smartphones	Dieperink et al [92]
Air pollution from cigarettes	Gunn et al [45]

a CO_2_: carbon dioxide.

b GHG: greenhouse gases.

c LCA: life cycle assessment.

### Measuring the Environmental Impact of DHTs

Assessment of travel-related carbon emissions was widely used, and software tools or applications were often used for distance calculations, particularly for determining round-trip travel distances [12,27,28,31,33,35-38,40,42,44-46,48,49,53-56,58,59,62,64-66,68-70,72,75,76,80,84-88,90]. The detailed list of software programs used is presented in Multimedia Appendix 5. In travel distance assessment, Google Maps was the most widely used application.

In the assessment of CO_2_ and GHG emissions, different published conversion factors or emissions calculators were used [12,27,28,30,32-34,36-38,40,42,43,45,47-59,61,62,64-68,71,72,75-77,81-85,87,88,90-93]. Similarly, in different studies that assess carbon emissions related to energy consumption [61,62,64,67,84], personal protective equipment [61], water usage and domestic waste [61], telecommunication [61], smartphone emission [48], desktop computer [48], electricity usage [39], published emission conversion, or calculators have been used. The detailed list of conversion factors or emissions calculators used is presented in Multimedia Appendix 5. For carbon footprint and GHG emission calculations, the widely used conversion factors and emission databases include the UK government (Department for Environment, Food and Rural Affairs and Department for Business, Energy and Industrial Strategy), the US Environmental Protection Agency (EPA), Transport Canada, the Greenhouse Gas Protocol, and country-specific emission factors from Australia, India, Brazil, Switzerland, Germany, and Canada and some online carbon calculators.

### Measurement Techniques Used in LCA

Several studies have used LCA to assess the carbon footprint of DHTs. Across studies, methodologies vary in scope, input data, and analytical frameworks, but all follow the principle of quantifying GHG emissions or carbon footprint across a product or service’s life cycle. Few studies have adopted the ISO 14040/14044 standards for LCA [73,79] and ISO 14060:2006 standards for quantification and reporting of GHGs [63]. This process includes 4 phases: goal and scope definition, life cycle inventory (LCI), life cycle impact assessment (LCIA), and interpretation [79]. The GHG Protocol, which includes Scopes 1‐3 emissions, has also been incorporated [63]. For example, in a study by Lathan et al [63], Scope 1 emissions include direct outputs such as anesthetic gases; Scope 2 includes purchased electricity; and Scope 3 includes indirect emissions such as water use, waste disposal, and patient travel.

In LCA, one of the common data sources used was the Ecoinvent database [29,39,41,79,86]. In life cycle impact assessment, several LCA software and databases were used, for example, SimaPro software [41,79], OpenLCA software [35], and FootprintCalc database [73]. To translate inventory data into environmental impact categories, environmental impact assessment methodologies, for example**,** ReCiPe 2016 [60] and Tool for Reduction and Assessment of Chemicals and Other Environmental Impacts (TRACI) 2.1 v1.06/ US 2008 [41] were used.

When analyzing carbon footprint, 2 approaches were used, such as process-based LCA [29,35,60,86,89] that include detailed activity and product-specific data, and Environmentally Extended Input-Output (EEIO) models [35,39,86]. When a specific product’s LCA is not available, this approach can be used. Some examples were Chinese EEIO (CEEIO) [86] and United States EEIO (USEEIO) [35]. In LCA, these 2 approaches were combined and assessed [35,39,86].

In LCA, different emission sources were used, for example, patient travel [29,35,63,86], infrastructure for telemedicine [29,89], medical equipment and consumables [29], laboratory and radiographic testing [35], medical sources, such as anesthetic gases [63], energy consumption [63,86], pharmaceutical data, such as antibiotics [63], and tariff data (staff cost and time) [63]. Additionally, different conversion factors were used to convert carbon-emission-related process and economic input data (Multimedia Appendix 5).

### Measurement Properties of the Carbon Footprint Assessment Tools and Techniques

The most reported measurement property was the sensitivity analysis [31,40,41,43,60,65,66,79,82,93,94] that focused on assessing the impact of assumptions on the results. The other approach, Monte Carlo Analysis (MCA), accounts for variability in the data by generating low and high estimates of possible GHG emissions for various input parameters [29,39,79].

### Strengths of Environmental Impact Assessment Methods and Techniques

Few researchers reported the strengths of environmental impact assessment methods. Comprehensive assessment of carbon footprint [69], including the broad span models [26] and LCA [39,41,63,73,86,89] was recognized. Within LCA, the application of input-output modeling [35] and the use of known economic information to estimate emissions for products without an LCA [29] were identified as strengths. In travel-related analyses, some studies included all travel modes, including new and follow-up visits [78,83], providing a more complete estimate of transport-related emissions. Additionally, some studies identified the inclusion of sensitivity analysis [31,79] as a strength of the carbon footprint assessment techniques.

### Limitations and Gaps of Environmental Impact Assessment Methods and Techniques

Many researchers have reported methodological limitations, such as single-center studies [28,33,35,38,71,88], small sample sizes [46,71], limited datasets [40,63], limited to one geographical area [34], and short study periods [84]. Another major limitation was reliance on retrospective data [35,38,40,43,44,52,62,76,80] or secondary data analysis [65] or reports from clinicians [48]. Data extraction from patients’ records posed additional challenges, including incomplete information and data errors [31], issues in data extraction, management, and cleaning [43], and inconsistencies in patient address records [93]. Design limitations included a modeling cohort design, which may have resulted in emission estimates that are not entirely representative [67]. Additionally, a lack of data quality assessment [73] and sensitivity analysis [73] was identified. Another reported limitation was the use of estimates. Some studies relied on validated calculators that may not reflect real-world variations in vehicle type, maintenance, fuel quality, or terrain [11,17,53], and carbon footprint and tree-planting calculations were approximations [58].

Another major limitation was making assumptions, particularly in calculating travel-related environmental impact. Such assumptions included that all journeys were made under normal conditions [64], in personal automobiles or private passenger vehicles (particularly cars) [27,28,34,43,45,47,49,50,52,53,59,64,68,81,85,90], with patients returning directly home after visits [27,33,54], all patients used either public transport or drove [58], and that all virtual visits replaced office visits [36] while excluding the aspects of public transport, large vehicles, or the use of diesel vehicles [47,54,62,80,81,90]. Other assumptions included that carbon emissions from the use of cars and taxis are equal [54] and are based on average emission values [62,78]. In some cases, travel distances were estimated based on midpoints rather than exact distances [43], on patient-reported data [82], or on the most recently recorded addresses in the EHR [44].

Some limitations related to the study scope were reported. Many studies assessed only one aspect of DHTs, particularly travel-related emissions, and it has been identified as a significant limitation [28,66,75,83]. Contributions from other sources include staff commuting [48,64,73], staff working remotely [60], and clinic maintenance, such as heating and supplies [66], paper use [76], or telemedicine infrastructure [51]. Some omitted electricity consumption during virtual consultations [45,50] or carbon emissions associated with devices such as cameras and cell phones [33], or equipment used at home, such as spirometers [85]. Other excluded aspects included follow-up in-person visits after teleconsultations [40,41], and broader factors such as road construction, maintenance, and parking [84], or emissions from other health care activities [36]. Other study scope-related limitations included only estimating CO_2_ emissions without accounting for other environmental pollutants [78], not investigating emissions produced by the hypothetical in-person office visits [27], and the carbon emissions savings reported were likely to be on the upper end of estimates [50].

Some limitations were reported related to LCA. Some calculations were limited to transport and electricity [86], telephone conferencing [34], or were based on simplified travel assumptions (eg, fixed distances and mode splits) [89]. These studies also excluded certain aspects, such as disposable items (eg, paper handouts, exam table sheets, and sanitizers) [28], the maintenance of hospital facilities [28], and fixed assets (eg, furniture, air conditioning, and network infrastructure) [86]. When using USEEIO models, inherent limitations remained, such as estimating GHG emissions based on the dollar spent in specific economic sectors, which may not fully reflect embodied emissions [35]. Furthermore, LCA data for videoconferencing peripherals were sometimes outdated or based on rough estimates [94], and the overall research base remains limited for the LCA studies [94].

A few additional limitations concerning different DHTs were reported. In remote monitoring, the relationship between monitoring frequency and GHG emissions or health care consumption was examined, focusing instead on pre-post comparisons [29]. In the assessment of energy consumption, the emission calculation was based on assumptions [70], and the equipment used for telecommunication (a computer, a webcam, and a loudspeaker) was similar for both patients and staff [84]. In relation to EHRs, related environmental impact assessment, impacts on built land, common air pollutants, and indirect or minor contributions were excluded, and only a narrow set of equivalences (eg, carbon sequestration acres) was reported [26].

### Environmental Impact of DHTs

Most studies reported a positive environmental impact of DHTs. These technologies contribute to environmental sustainability and reducing climate change by supporting greener models of health care delivery and advancing net-zero goals [12,32,37,42,53,54,57,80,84,87]. Telehealth and telemedicine interventions, including virtual clinics and telerehabilitation, have demonstrated substantial potential in reducing health and social care’s carbon footprint. These interventions significantly reduced patient and/or staff travel [12,27-29,31-34,36,39,41-44,46-59,61,62,64,66,68,69,71-74,76-78,80,81,83,84,86-88,90-92,94] and fuel reduction [27,33,42,80,81], resulting in positive environmental impacts through a reduction in carbon footprint [12,14,36,37,39,41,52-55,58,59,61,62,64,65,67,73,83,84,86,87,91,93,94], CO_2_ emissions [27,28,30,32,34,38,40,44,47-51,56,57,66,68,71,72,75-78,81,88,90,92], GHG emissions [31,33,42,43,45,80,82] or other air pollutant emissions [31,45,64,80,82]. Energy consumption [49] for video conferencing [64], phone call [41,54], software design and programming [86], electronic products, including video equipment [86] and computers [41], data use [61], and staff travel [61] were the identified contributors to the environmental impact of the above DHTs.

Other virtual platforms, such as remote monitoring systems, have shown potential in reducing carbon footprint [63,84]. An eHealth system, including e-referral and e-prescription services, was reported to potentially reduce carbon footprint by up to 50% through decreased travel [89]. In this study, approximately 90% of the remaining environmental impact was attributed to the use of computers and network infrastructure [89]. In another study, a digital wound management platform demonstrated positive environmental impacts by reducing travel and CO_2_ emissions [70].

Turley et al [26] revealed that, despite higher energy consumption, EHRs have a positive net effect on the environment. According to this study, EHRs reduced paper consumption, transportation, plastic waste from X-rays, toxic chemicals, and water usage, but energy-intensive data centers and hardware increased the carbon footprint considerably [26]. A digital health and well-being service that included 6 electronic devices was reported to have a considerable environmental carbon footprint (718‐1509 kg CO_2_e per resident), with electricity consumption identified as the main contributing factor [60]. A digital health app that included video consultations was found to contribute to reduced patient travel and GHG, and other pollutants [82].

While digital health technologies are often associated with reductions in carbon emissions, several studies have also reported potential negative environmental impacts. Electricity consumption was identified as the principal contributor to the overall environmental footprint [60], with the energy required to power computers and mobile devices remaining substantial [41]. Kwon et al [79] reported that EHR systems emitted significantly more GHGs than paper-based systems, with 90% of emissions arising from electricity use to run the system.

Tarpani and Gallego-Schmid [60] reported a considerable negative environmental impact from a digital health and well-being service that included six electronic devices, resulting in a CO_2_ equivalent of 718‐1509 kg per person, and identified electricity as the main contributor. Tarpani and Gallego-Schmid [60] highlighted that if not properly managed, the rapid expansion of digitalization could intensify these impacts, resulting in higher energy demands that lead to climate change and increased ecotoxicity from the mining and processing of metals, often sourced from developing countries.

## Discussion

### Principal Findings

This scoping review provides a comprehensive overview of the current evidence on the environmental impact of DHTs across different health care settings, including assessment methods and their strengths and limitations. We synthesized the findings from studies conducted from 2007 to 2025. Overall, the evidence suggests that DHTs have the potential to reduce environmental impacts, particularly through decreasing patient and clinician travel, reducing paper consumption, and improving the efficiency of health care delivery. However, the environmental benefits were not consistently reported across studies, and negative impacts were identified regarding the energy consumption, digital infrastructure requirements, and electronic waste associated with the implementation of the DHTs. Most published studies were conducted in high-income countries, which limits the direct generalizability of these findings to low- and middle-income countries (LMICs) with different health system infrastructures and energy grid emission factors, indicating a need to expand assessments of the environmental impact of DHTs in LMICs. Most studies have been conducted in primary or tertiary care settings, highlighting the need to expand research into other settings, such as older age care and social services. Additionally, considerable variation was observed in the methods used to assess environmental impacts, limiting the comparability of findings and highlighting the need for more standardized assessment approaches.

Methodological quality of the included studies appeared to vary considerably. Most studies were retrospective or secondary analyses based on routinely collected data, and only a few were prospective. Many studies reported several limitations, including small sample sizes, single-site evaluations or small-scale studies, reliance on assumptions regarding travel avoidance, reliance on estimates in carbon footprint assessments, and incomplete LCAs of technologies. Moreover, substantial methodological heterogeneity was observed, preventing direct comparisons of findings across studies and suggesting that quantitative meta-analysis would not be feasible in future systematic reviews on the topic. The included studies [12,26-94] vary considerably in the environmental outcomes assessed, measurement approaches, assumptions, and reporting methods. Additionally, these methodological constraints in assessing the environmental impact of DHTs may influence the interpretation of findings, thereby limiting the accuracy of reported environmental impacts. Therefore, the findings should be interpreted with caution. Although available evidence supports reducing the carbon footprint, the magnitude of these benefits remains uncertain. Therefore, the findings of this review should be interpreted as an overview of the current evidence landscape rather than definitive estimates of the environmental benefits of DHTs.

This review reveals that most included studies evaluate the environmental impact of virtual interventions, specifically telehealth and telemedicine services, delivered via video, telephone, or hybrid models, compared to other DHTs, for example, AI, mHealth applications, and IoT technologies, indicating an important finding of this scoping review. Following the COVID-19 pandemic, the adoption of telehealth and telemedicine increased substantially [95]. Most studies have examined the environmental impact of virtual interventions during or after the COVID-19 pandemic. Additionally, assessing the environmental impact of virtual interventions is relatively straightforward because they are associated with direct, measurable environmental benefits, particularly through reductions in patient and clinician travel. In contrast, the assessment of environmental impacts of other DHTs may be more complex as it involves indirect effects, energy consumption, data processing, digital infrastructure, and lifecycle considerations. The lack of standardized and validated methods and tools for assessing the environmental impact of digital health care is also a significant drawback [96]. As our review covered the literature from inception, it may overrepresent virtual interventions compared with other emerging or relatively newer DHTs, such as mHealth applications, IoT technologies, and AI, for which the evidence base is less mature. Additionally, following the COVID-19 pandemic, many virtual interventions have been introduced. Consequently, current evidence supporting environmental benefits is largely derived from telemedicine and telehealth, and caution is warranted when generalizing these findings to DHTs more broadly. However, recent reviews indicate that telemedicine has emerged as a promising solution to reduce the carbon footprint of health care [16] and highlight its ongoing importance in advancing sustainable health care delivery [17,94]. Other DHTs, including EHRs, eHealth and mHealth applications, AI interventions, and robotics, have been rarely or not studied, highlighting the need to extend environmental assessments to these technologies.

Most studies employed travel-related carbon-emission assessments, reflecting the link between reduced patient and staff travel and lower GHG emissions, consistent with the previous findings [16,97]. These analyses were often based on software that calculates distance and published conversion factors. More comprehensive approaches, such as LCA, including process-based and EEIO models, were less commonly used. These approaches provide a more holistic picture of emissions from infrastructure, travel, energy use, medical consumables, and information technology systems used in DHTs. LCA offers the most comprehensive approach for quantifying carbon emissions and enables detailed analysis [98]. The use of established standards, for example, ISO 14040/14044, and recognized databases, such as Ecoinvent and SimaPro in some studies, strengthens the validity and reproducibility of the findings. Some studies have expressed CO_2_ emissions using CO_2_ emission equivalencies, such as equivalent carbon sequestration and equivalent CO_₂_ emissions, providing a more relatable way to communicate environmental impact. Reporting carbon footprint information in more familiar terms, such as everyday metrics or equivalencies, can improve communication and public understanding, which, in turn, may support interventions aimed at promoting more sustainable behaviors [99], adding a strength to carbon footprint assessment approaches. Moreover, some researchers have employed sensitivity analysis to address uncertainties, thereby enhancing the reliability of their findings. However, there was considerable heterogeneity in methodologies, with many studies focusing solely on travel-related environmental impacts while overlooking broader factors such as energy consumption, manufacturing, waste generation, and disposal, thereby presenting a major limitation in environmental assessment approaches.

This review identified several methodological limitations in environmental impact assessment methods. LCA studies faced additional challenges due to inconsistencies in methodological approaches, including differences in the system boundaries adopted, with some studies assessing only selected stages of an intervention or technology while others evaluated impacts across the entire life cycle. Furthermore, studies used different emission factors and data sources to estimate environmental impacts. These methodological differences limited the comparability of findings across studies and contributed to the substantial heterogeneity that prevented quantitative synthesis. Many studies relied on retrospective or secondary data, small sample sizes, or single-center designs, which limited the generalizability of their findings. Travel-related analyses often involved broad assumptions, such as uniform vehicle types, direct journeys, or complete substitution of in-person visits, that may have resulted in over- or underestimation of environmental benefits. Most travel-based environmental impact assessments assumed that virtual consultations would be replaced by face-to-face visits. However, this assumption may not always reflect real-world practice. This approach may overlook induced demand, whereby telemedicine may increase the total number of consultations, and rebound effects, whereby time saved may be spent on other carbon-intensive activities. Therefore, the environmental benefits reported in some studies may have been overestimated.

Moreover, studies frequently considered only one or a few aspects, while significant contributors, such as staff commuting, clinic infrastructure, and follow-up visits, were often overlooked. LCAs face additional challenges, including reliance on secondary or retrospective data, limited consideration of indirect emissions, and the use of monetary proxies in EEIO models. Consistent with recent literature [96,100], these gaps underscore the need for more rigorous and standardized frameworks to assess the environmental impact of DHTs accurately.

Researchers have paid limited attention to reporting the measurement properties of carbon footprint assessment methods. The most frequently reported measurement properties were sensitivity analysis and MCA. In environmental impact assessment, model-based approaches are commonly used rather than direct measurement; therefore, traditional psychometric concepts such as validity and reliability are less applicable. Current assessment approaches have focused on evaluating the robustness and uncertainty of carbon footprint estimates. As few studies report these types of analyses, greater attention should be given to systematically assessing uncertainty and robustness in future research to improve the reliability and transparency of carbon footprint assessments.

This scoping review suggests that DHTs have a positive environmental impact. Previous literature reviews [15,16,96] also reported positive environmental impact on DHTs. However, estimates varied substantially according to DHT type, health care setting, geographic location, carbon footprint assessment methods, system boundaries, and underlying assumptions. Additionally, environmental impacts were reported using diverse metrics, including travel distance avoided, travel-related emissions savings, CO_₂_ emissions, GHG emissions, carbon footprint estimates, and CO_₂_ emission equivalencies. This variability limited direct comparisons between studies and highlighted a lack of consistency in environmental impact assessment approaches. Telemedicine interventions bring positive environmental impacts primarily through reductions in patient travel and associated GHG emissions. These reported benefits align with broader sustainability objectives, particularly supporting the net-zero goals of national health systems in many countries.

However, very few studies reported negative impacts, including high electricity consumption for powering devices and ICT infrastructure, as well as environmental burdens from the production and disposal of digital equipment. For example, Kwon et al [79] reported 90% of the GHG emissions associated with EHR systems arose from electricity consumption, while Tarpani et al [60] reported a carbon footprint of 718‐1509 kg CO_2_e per person for a digital health and well-being service incorporating multiple electronic devices. These findings indicate that negative environmental impacts may be considerable for some DHTs, particularly those that depend on continuous electricity consumption, extensive digital infrastructure, or multiple interconnected devices. Without careful management, these factors could undermine the positive environmental impact of DHTs and limit the net environmental benefits of digital health care interventions, particularly those stemming from reduced travel and more efficient resource use. Therefore, the environmental benefits of DHTs should be interpreted with caution, as the available evidence is characterized by considerable negative impacts, possible underreporting of these impacts, methodological heterogeneity, and the use of diverse assessment approaches. Therefore, more robust conclusions about the environmental sustainability of DHTs require comprehensive LCAs that use standardized, validated assessment approaches.

### Strengths and Limitations of the Study

This scoping review has several strengths that enhance the reliability and comprehensiveness of its findings on the environmental impact of DHTs across different health care settings. We followed a transparent and robust review process, adhering to established guidelines and best practices, including the scoping review framework proposed by Arksey and O’Malley [23] and the PRISMA-ScR guidelines [24]. We included studies from inception to the present, ensuring that no study examining the environmental impact of DHTs was overlooked. Additionally, reviewing reference lists helped identify further relevant studies.

However, this review also has some limitations. The search was limited to 3 databases and to English-language papers, which may have resulted in the exclusion of relevant studies. We did not include gray literature, which may have led to the omission of evaluations conducted by governments or private-sector organizations. Governments and private-sector organizations often report environmental impact assessments; therefore, the current review may not have captured the full breadth of relevant evidence. However, we searched 3 broad databases without time limitations, which allowed us to capture a range of peer-reviewed studies. Moreover, we observed substantial heterogeneity across the included studies; therefore, although additional evidence may provide further insights, it is unlikely to substantially change the overall findings of this review. Additionally, we did not conduct a quality appraisal in this scoping review, which may be considered a limitation. However, given the broad objectives of this study, we included studies based on the value of the information they provided. Moreover, the literature suggests that quality appraisal is not mandatory in scoping reviews due to the nature and purpose of the review type [25].

### Implications for Practice, Research, and Policy

This scoping review suggests several recommendations to strengthen practice, research, and policy related to the environmental impact of DHTs. For practice, health care providers adopting DHTs should consider both clinical and environmental outcomes when designing and scaling interventions. Virtual care programs may be expanded to help reduce the carbon footprint in health and social care [53,67,80]. However, overall environmental impact is complex, as potential reductions from avoided travel may be offset by increased energy consumption, use of digital infrastructure, and the lifecycle impacts of devices used to deliver virtual care. Therefore, the implementation of such interventions requires careful consideration of both positive and negative environmental impacts, along with a cost-benefit analysis. Exploring the potential of telehealth to promote health equity by improving access and reducing environmental impact is crucial [45]. More comprehensive LCAs are needed to assess the true environmental impact of DHTs that inform long-term sustainability planning.

For research, expanding evaluation frameworks [62] and developing and validating standardized methodologies, incorporating novel approaches [64,65,86] for carbon footprint assessment of DHTs, are essential. Conducting large-scale studies to validate findings [63,94] and collecting prospective data to capture detailed and accurate data [63] are recommended. The use of larger, more diverse, multicenter samples improves the generalizability of findings. As the majority of studies focus on assessing travel-related carbon footprint, expanding environmental impact assessments to include other aspects, for example, energy use, supply chain emissions, and waste disposal, is recommended [73]. Carbon footprint assessments in the community and social care sector should be strengthened. Given that digitalization often depends on resource-intensive supply chains in developing countries, the social implications of deploying digital health services [60], particularly with a focus on equity and global justice, are emphasized.

From policy perspectives, this review highlights the potential of DHTs as a critical component of sustainable health care strategies. Securing political support for digital health implementation [85], adopting energy-efficient technologies [77], and ensuring the integration of environmental considerations into technology assessments and economic evaluations [91] are some key strategies for promoting sustainable digital health. Policymakers and health care organizations need to acknowledge the trade-offs of digitalization and prioritize investments in green digital infrastructure.

### Conclusion

Most research is conducted in high-income countries and focuses on telehealth and telemedicine, primarily assessing travel-related environmental impacts and GHG emissions. Different approaches are employed to measure environmental impact. Direct environmental assessment includes the assessment of travel, CO_2_ and GHG emissions, and LCA. In LCA, both process-based and EEIO models are incorporated. Another approach is expressing CO_₂_ emissions using CO_₂_ emission equivalencies, such as equivalent carbon sequestration and equivalent CO_₂_ emissions, providing a more relatable way to communicate environmental impact. There is limited evidence regarding the measurement properties of environmental impact assessment techniques. The available evidence, predominantly derived from telemedicine studies, suggests that DHTs may contribute to environmental benefits, including reductions in travel and carbon emissions, under certain conditions. However, these findings should be interpreted cautiously given the substantial methodological heterogeneity identified across studies, reliance on different assumptions, limited evidence relating to other types of DHTs, and potential negative environmental impacts associated with electricity consumption, digital infrastructure, and device life cycles. Additionally, the methodological quality of the evidence appeared to vary considerably. Consequently, the magnitude of these environmental benefits should be further interpreted with caution. To better understand the environmental implications of DHTs, more rigorous, standardized, validated, and comprehensive assessment methods are required. Bridging these methodological gaps is essential to ensure that DHTs not only support sustainable health care delivery but also make a meaningful contribution to mitigating global climate change.

## Supplementary material

10.2196/88341 Multimedia Appendix 1 Keywords and MeSH terms.

10.2196/88341 Multimedia Appendix 2 Search strategy.

10.2196/88341 Multimedia Appendix 3 Excluded studies.

10.2196/88341 Multimedia Appendix 4 Study characteristics.

10.2196/88341 Multimedia Appendix 5 Software programs and conversion factors or emissions calculators used in environmental impact assessment.

10.2196/88341 Checklist 1 PRISMA-ScR checklist.

## Abbreviations

CEEIO: Chinese Environmentally Extended Input-Output
CH_4_: methane
CO_2_: carbon dioxide
CO_2_e: carbon dioxide equivalent
DHT: digital health care technology
EEIO: Environmentally Extended Input-Output
EHR: electronic health record
EPA: Environmental Protection Agency
GHG: greenhouse gas
IoT: Internet of Things
ISO: International Standard Organization
LCA: life cycle assessment
LCI: life cycle inventory
LCIA: life cycle impact assessment
LMIC: low-and middle-income country
MCA: Monte Carlo Analysis
mHealth: mobile health
N_2_O: nitrous oxide
PRISMA-ScR: Preferred Reporting Items for Systematic Reviews and Meta-Analyses extension for Scoping Reviews
TRACI: Tool for Reduction and Assessment of Chemicals and Other Environmental Impacts
USEEIO: United States Environmentally Extended Input-Output
VA: Veterans Affairs

## References

[1] (2025). Climate change. World Health Organization.

[2] (2023). AR6 synthesis report-climate change 2023. https://www.ipcc.ch/report/ar6/syr.

[3] (2023). A guide to carbon footprinting for businesses. Carbon Trust.

[4] Wiedmann T, Minx J (2008). A definition of ‘carbon footprint’. https://wiki.epfl.ch/hdstudio/documents/articles/a%20definition%20of%20carbon%20footprint.pdf.

[5] Huang J, Chen X, Zhao X (2024). How digital technology reduces carbon emissions: from the perspective of green innovation, industry upgrading, and energy transition. J Knowl Econ.

[6] Usher K, Williams J, Jackson D (2024). The potential of virtual healthcare technologies to reduce healthcare services’ carbon footprint. Front Public Health.

[7] (2019). Healthcare climate’s climate footprint; how the health sector contributes to the global climate crisis and opportunities for action. https://global.noharm.org/sites/default/files/documents-files/5961/HealthCaresClimateFootprint_092319.pdf.

[8] Alotaibi N, Wilson CB, Traynor M (2025). Enhancing digital readiness and capability in healthcare: a systematic review of interventions, barriers, and facilitators. BMC Health Serv Res.

[9] (2021). Global strategy on digital health 2020-2025. https://www.who.int/docs/default-source/documents/gs4dhdaa2a9f352b0445bafbc79ca799dce4d.pdf.

[10] Kaihlanen AM, Laukka E, Nadav J (2023). The effects of digitalisation on health and social care work: a qualitative descriptive study of the perceptions of professionals and managers. BMC Health Serv Res.

[11] Faizan M, Han C, Lee SW (2025). Policy-driven digital health interventions for health promotion and disease prevention: a systematic review of clinical and environmental outcomes. Health Care (Don Mills).

[12] Marković S, Kralj D, Knežević Ivanovski T, Svorcan P (2025). Telehealth-an environmentally friendly way to take care of patients with inflammatory bowel disease. Medicina (Kaunas).

[13] Forslund M, Mathieson K, Djibo Y, Mbindyo C, Lugangira N, Balasubramaniam P (2024). Strengthening the evidence base on the use of digital health technologies to accelerate progress towards universal health coverage. Oxf Open Digit Health.

[14] Wang X, Su X (2025). Does digitalization facilitate pollution and carbon emissions reduction synergies?: Evidence based on Chinese A-share manufacturing companies. Environ Dev Sustain.

[15] Purohit A, Smith J, Hibble A (2021). Does telemedicine reduce the carbon footprint of healthcare? A systematic review. Future Healthc J.

[16] van der Zee C, Chang-Wolf J, Koopmanschap MA, van Leeuwen R, Wisse RP (2024). Assessing the carbon footprint of telemedicine: a systematic review. Health Serv Insights.

[17] Plath J, Dziggel TF, Karpa DF, Lange O, Runge T, Rogowski WH (2021). The carbon footprint of digital health interventions: the development of a transparency catalogue and a systematic review of the evidence. SSRN.

[18] Rodler S, Ramacciotti LS, Maas M (2023). The impact of telemedicine in reducing the carbon footprint in health care: a systematic review and cumulative analysis of 68 million clinical consultations. Eur Urol Focus.

[19] Smith A, Severn M (2023). Reducing the environmental impact of clinical care. Can J Health Technol.

[20] Al Amosh H, Khatib SFA (2025). Environmental innovation and carbon emissions reduction in European healthcare: the moderating role of environmental monitoring practices. Clean Respons Consum.

[21] Nissinen A, Pitkänen A, Barchuk A (2025). The climate impacts of healthcare digitalization: a scoping review. Digit Health.

[22] (2022). ISO 14040: environmental management-life cycle assessment-principles and framework. International Organization for Standardization.

[23] Arksey H, O’Malley L (2005). Scoping studies: towards a methodological framework. Int J Soc Res Methodol.

[24] Tricco AC, Lillie E, Zarin W (2018). PRISMA Extension for Scoping Reviews (PRISMA-ScR): checklist and explanation. Ann Intern Med.

[25] Alexander L, Aromataris E, Barber B (2024). JBI Manual for Evidence Synthesis.

[26] Turley M, Porter C, Garrido T (2011). Use of electronic health records can improve the health care industry’s environmental footprint. Health Aff.

[27] Danesh DO, Luca J, Kalady SE, Townsend JA, Hammersmith KJ, Meyer BD (2025). The impact of teledentistry on travel distance and carbon emissions at a children’s hospital. J Am Dent Assoc.

[28] Kellerman H, Mace JC, Detwiller KY, Geltzeiler M, Smith TL, Pandrangi VC (2025). Travel costs and carbon savings associated with telemedicine in a tertiary care rhinology center. Int Forum Allergy Rhinol.

[29] Martinez KA, Haider A, Tarefder R, Khan Z, Meiklejohn DA, Zhu J (2025). Piloting total perioperative telemedicine for elective cholecystectomies for symptomatic cholelithiasis. Surg Endosc.

[30] Wong V, Cohen J, Ingram A (2025). Patient-centered cost saving and positive environmental impact with the introduction of telehealth services at a single center. Urol Pract.

[31] Dullet NW, Geraghty EM, Kaufman T (2017). Impact of a university-based outpatient telemedicine program on time savings, travel costs, and environmental pollutants. Value Health.

[32] Mojdehbakhsh RP, Rose S, Peterson M, Rice L, Spencer R (2021). A quality improvement pathway to rapidly increase telemedicine services in a gynecologic oncology clinic during the COVID-19 pandemic with patient satisfaction scores and environmental impact. Gynecol Oncol Rep.

[33] Roy W, Hans B, Jannat RU, Reddy YK, Hadi Y, Gayam S (2023). Tele-visits for GERD: “Ecofriendly, efficient and effective”. J Gastroenterol Hepatol.

[34] Robinson JD, Prochaska JD, Yngve DA (2017). Pre-surgery evaluations by telephone decrease travel and cost for families of children with cerebral palsy. SAGE Open Med.

[35] Wang EY, Zafar JE, Lawrence CM (2021). Environmental emissions reduction of a preoperative evaluation center utilizing telehealth screening and standardized preoperative testing guidelines. Resour Conserv Recycl.

[36] Dacones I, Cave C, Furie GL, Ogden CA, Slutzman JE (2021). Patient transport greenhouse gas emissions from outpatient care at an integrated health care system in the Northwestern United States, 2015–2020. J Clim Chang Health.

[37] Whetten J, Montoya J, Yonas H (2019). Access to better health and clear skies: telemedicine and greenhouse gas reduction. Telemed J E Health.

[38] Thota R, Gill DM, Brant JL, Yeatman TJ, Haslem DS (2020). Telehealth Is a sustainable population health strategy to lower costs and increase quality of health care in rural Utah. JCO Oncol Pract.

[39] Sillcox R, Gitonga B, Meiklejohn DA (2023). The environmental impact of surgical telemedicine: life cycle assessment of virtual vs. in-person preoperative evaluations for benign foregut disease. Surg Endosc.

[40] Iaccarino MA, Paganoni S, Tenforde A (2022). Environmental impact of telerehabilitation visits in an urban setting. J Clim Chang Health.

[41] Thiel CL, Mehta N, Sejo CS (2023). Telemedicine and the environment: life cycle environmental emissions from in-person and virtual clinic visits. NPJ Digit Med.

[42] Moore SL, Grim S, Kessler R, Luna MLD, Miller DE, Thomas JF (2024). Reduction of environmental pollutants and travel burden through an academic medical center-based electronic consultation program. Telemed J E Health.

[43] Sharma S, Yellowlees PM, Gotthardt CJ, Luce MS, Avdalovic MV, Marcin JP (2023). Environmental impact of ambulatory telehealth use by a statewide university health system during COVID-19. Telemed J E Health.

[44] Cockrell HC, Maine RG, Hansen EE (2022). Environmental impact of telehealth use for pediatric surgery. J Pediatr Surg.

[45] Gunn AH, Murray EM, Patel MR, Mentz RJ (2024). Carbon emissions and air pollution savings among telehealth visits for cardiology appointments. Am Heart J Plus.

[46] Jiang CY, Strohbehn GW, Dedinsky RM (2021). Teleoncology for veterans: high patient satisfaction coupled with positive financial and environmental impacts. JCO Oncol Pract.

[47] Sillcox R, Blaustein M, Khandelwal S, Bryant MK, Zhu J, Chen JY (2023). Telemedicine use decreases the carbon footprint of the bariatric surgery preoperative evaluation. Obes Surg.

[48] Penaskovic KM, Zeng X, Burgin S, Sowa NA (2022). Telehealth: reducing patients’ greenhouse gas emissions at one academic psychiatry department. Acad Psychiatry.

[49] Cummins MR, Shishupal S, Wong B (2024). Travel distance between participants in US telemedicine sessions with estimates of emissions savings: observational study. J Med Internet Res.

[50] Patel KB, Gonzalez BD, Turner K (2023). Estimated carbon emissions savings with shifts from in-person visits to telemedicine for patients with cancer. JAMA Netw Open.

[51] Grabski DF, Meyer MJ, Gander JW (2024). Pediatric telemedicine visits reduce greenhouse gas emissions. J Clim Chang Health.

[52] Moore L, Balmer F, Woywodt A (2024). The environmental impact of changing to virtual renal transplant aftercare: 2-year experience with a single outpatient clinic. Future Healthc J.

[53] Miah S, Dunford C, Edison M (2019). A prospective clinical, cost and environmental analysis of a clinician-led virtual urology clinic. Ann R Coll Surg Engl.

[54] King J, Poo SX, El-Sayed A (2023). Towards NHS zero: greener gastroenterology and the impact of virtual clinics on carbon emissions and patient outcomes. A multisite, observational, cross-sectional study. Frontline Gastroenterol.

[55] Bhavanasi BA, Kulkarni S (2024). Impact of virtual clinics on streamlining NHS outpatient waiting lists and carbon emissions. Foot Ankle Orthop.

[56] Dorrian C, Ferguson J, Ah-See K (2009). Head and neck cancer assessment by flexible endoscopy and telemedicine. J Telemed Telecare.

[57] Lewis D, Tranter G, Axford AT (2009). Use of videoconferencing in Wales to reduce carbon dioxide emissions, travel costs and time. J Telemed Telecare.

[58] Connor MJ, Miah S, Edison MA (2019). Clinical, fiscal and environmental benefits of a specialist-led virtual ureteric colic clinic: a prospective study. BJU Int.

[59] Sellars H, Ramsay G, Sunny A, Gunner CK, Oliphant R, Watson AJM (2020). Video consultation for new colorectal patients. Colorectal Dis.

[60] Tarpani RRZ, Gallego-Schmid A (2024). Environmental impacts of a digital health and well-being service in elderly living schemes. Clean Environ Syst.

[61] Bartlett S, Keir S (2022). Calculating the carbon footprint of a geriatric medicine clinic before and after COVID-19. Age Ageing.

[62] Blenkinsop S, Foley A, Schneider N, Willis J, Fowler HJ, Sisodiya SM (2021). Carbon emission savings and short-term health care impacts from telemedicine: an evaluation in epilepsy. Epilepsia.

[63] Lathan R, Hitchman L, Walshaw J (2024). Telemedicine for sustainable postoperative follow-up: a prospective pilot study evaluating the hybrid life-cycle assessment approach to carbon footprint analysis. Front Surg.

[64] Masino C, Rubinstein E, Lem L, Purdy B, Rossos PG (2010). The impact of telemedicine on greenhouse gas emissions at an academic health science center in Canada. Telemed J E Health.

[65] Mainer-Pearson G, Stewart K, Williams K (2025). Estimating patient and family costs and CO2 emissions for telehealth and in-person health care appointments in British Columbia, Canada: geospatial mixed methods study. J Med Internet Res.

[66] Galts C, Anvari S, Kim A, Leontiadis G, Armstrong D (2025). Sustainable practice in gastroenterology: travel-related CO_2_ emissions for gastroenterology clinic appointments in Canada. J Can Assoc Gastroenterol.

[67] Heffernan A, Lalande A, Chadha R, MacNeill A, Chadha NK (2024). Carbon savings potential of virtual care in obstructive sleep apnea and otitis media with effusion. Laryngoscope Investig Otolaryngol.

[68] Lambert P, Musto G, Thiessen M, Czaykowski P, Decker K (2023). Impact of cancer-related virtual visits on travel distance, travel time, and carbon dioxide (CO_2_) emissions during the COVID-19 pandemic in Manitoba, Canada. Curr Oncol.

[69] Smith AC, Kimble RM, O’brien A, Mill J, Wootton R (2007). A telepaediatric burns service and the potential travel savings for families living in regional Australia. J Telemed Telecare.

[70] Barakat-Johnson M, Kita B, Jones A (2022). The viability and acceptability of a virtual wound care command centre in Australia. Int Wound J.

[71] Schulz TR, Kanhutu K, Sasadeusz J, Watkinson S, Biggs BA (2020). Using telehealth to improve access to hepatitis C treatment in the direct-acting antiviral therapy era. J Telemed Telecare.

[72] Andrew N, Barraclough KA, Long K (2020). Telehealth model of care for routine follow up of renal transplant recipients in a tertiary centre: a case study. J Telemed Telecare.

[73] Savoldelli A, Landi D, Rizzi C (2024). Exploring the environmental impact of telemedicine: a life cycle assessment. Stud Health Technol Inform.

[74] Materazzo M, Facchini A, Garozzo D (2022). Maintaining good practice in breast cancer management and reducing the carbon footprint of care: study protocol and preliminary results. World Cancer Research J.

[75] Papavero SC, Fracasso A, Ramaglia P, Cicchetti A, de Belvis AG, Ferrara FM (2023). Telemedicine has a social impact: an Italian national study for the evaluation of the cost-opportunity for patients and caregivers and the measurement of carbon emission savings. Telemed J E Health.

[76] Desenzi Ciaralo PP, Guerreiro Cardoso PF, Minamoto H, Bibas BJ, Ribeiro de Carvalho CR, Pego-Fernandes PM (2025). Estimated carbon emissions and support cost savings to telemedicine for patients with tracheal diseases. Int J Med Inform.

[77] Prosperi Desenzi Ciaralo P, Guerreiro Cardoso PF, Minamoto H, Bibas BJ, Ribeiro de Carvalho CR, Pego-Fernandes PM (2024). Implementation and results of a dedicated telemedicine program (TeleTrachea) for patients with tracheal diseases. Telemed J E Health.

[78] Rani PK, Khanna RC, Ravindrane R (2024). Teleophthalmology at a primary and tertiary eye care network from India: environmental and economic impact. Eye (Lond).

[79] Kwon C, Essayei L, Spencer M (2024). The environmental impacts of electronic medical records versus paper records at a large eye hospital in India: life cycle assessment study. J Med Internet Res.

[80] Vidal-Alaball J, Franch-Parella J, Lopez Seguí F, Garcia Cuyàs F, Mendioroz Peña J (2019). Impact of a telemedicine program on the reduction in the emission of atmospheric pollutants and journeys by road. Int J Environ Res Public Health.

[81] Maria MS, Silvia AN, Beatriz DG, Andrew D, Guillermo PF (2022). Health care in rural areas: proposal of a new telemedicine program assisted from the reference health centers, for a sustainable digitization and its contribution to the carbon footprint reduction. Heliyon.

[82] Muschol J, Heinrich M, Heiss C (2022). Economic and environmental impact of digital health app video consultations in follow-up care for patients in orthopedic and trauma surgery in Germany: randomized controlled trial. J Med Internet Res.

[83] Arndt EM, Jansen TR, Bojko J (2023). COVID-19 measures as an opportunity to reduce the environmental footprint in orthopaedic and trauma surgery. Front Surg.

[84] Filfilan A, Anract J, Chartier-Kastler E (2021). Positive environmental impact of remote teleconsultation in urology during the COVID-19 pandemic in a highly populated area. Prog Urol.

[85] Oppelaar MC, Bannier M, Reijers MHE (2024). Remote monitoring and teleconsultations can reduce greenhouse gas emissions while maintaining quality of care in cystic fibrosis. Front Digit Health.

[86] Li Z, Xiang L, Ning J, Li W, Huang Y, Xiao X (2025). Pathways to sustainable health care development: study on the carbon reduction potential of telemedicine in China. J Med Internet Res.

[87] Özen F, Kaynar AH, Korkut AK, Teker Açıkel ME, Kaynar ZD, Kaynar AM (2024). The role of telemedicine towards improved sustainability in healthcare and societal productivity in Turkey. PLoS One.

[88] Meryk A, Salvador C, Kropshofer G (2025). Pioneering sustainable treatment delivery in childhood leukemia through synchronous telemedicine-a pilot study. Int J Cancer.

[89] Giacomo P, Håkansson P (2011). A Method to Measure the Reduction of CO 2 Emissions in E-Health Applications, User Centred Networked Health Care.

[90] Croghan SM, Rohan P, Considine S (2021). Time, cost and carbon-efficiency: a silver lining of COVID era virtual urology clinics?. Ann R Coll Surg Engl.

[91] Schmitz-Grosz K, Sommer-Meyer C, Berninger P (2023). A telemedicine center reduces the comprehensive carbon footprint in primary care: a monocenter, retrospective study. J Prim Care Community Health.

[92] Dieperink KB, Vestergaard LV, Møller PK, Tolstrup LK (2023). Using video consultations for clinical assessment and decision of treatment readiness before chemotherapy: a mixed-methods study among patients with gastrointestinal cancer and oncology nurses. Digit Health.

[93] Oliveira TC, Barlow J, Gonçalves L, Bayer S (2013). Teleconsultations reduce greenhouse gas emissions. J Health Serv Res Policy.

[94] Holmner A, Ebi KL, Lazuardi L, Nilsson M (2014). Carbon footprint of telemedicine solutions-unexplored opportunity for reducing carbon emissions in the health sector. PLoS One.

[95] Bouabida K, Lebouché B, Pomey MP (2022). Telehealth and COVID-19 pandemic: an overview of the telehealth use, advantages, challenges, and opportunities during COVID-19 pandemic. Health Care (Don Mills).

[96] Lokmic-Tomkins Z, Davies S, Block LJ (2022). Assessing the carbon footprint of digital health interventions: a scoping review. J Am Med Inform Assoc.

[97] Tennison I, Roschnik S, Ashby B (2021). Health care’s response to climate change: a carbon footprint assessment of the NHS in England. Lancet Planet Health.

[98] Li Y, Zha Y, Li M, Qian L (2025). Evaluating the carbon footprint of a hospital from the dynamic life cycle perspective: a case study in Shanghai. Build Environ.

[99] Filipowicz ALS, Shamma DA, Mohanty V, CL H (2025). Demystifying CO2: lessons from nutrition labeling and step counting. arXiv.

[100] Williams JTW, Bell KJL, Morton RL, Dieng M (2024). Methods to include environmental impacts in health economic evaluations and health technology assessments: a scoping review. Value Health.

