# Supplementary material for: Environmental Impact of Digital Health Care Technologies in Reducing Carbon Footprints: Scoping Review
Source: J Med Internet Res. 2026 Oct 21;28:e88341. doi: 10.2196/88341 (PMC13717332; doi:10.2196/88341)
Supplement: Supplementary file 1 [file jmir-v28-e88341-s001.docx]

**Appendix 1: Keywords and Mesh terms**

| Digital Healthcare Technologies | Health and Care | Carbon Footprint |
| --- | --- | --- |
| **Mesh terms**  Digital Health OR Telemedicine OR Electronic Health Records OR Decision Support System  **Keywords**  Electronic Health OR Electronic Medical Record* OR EMR OR EHR OR Digital Health OR e-Health OR m-Health OR DHT OR Clinical Decision Support System OR CDSS OR Telemedicine OR Telenursing | **Mesh terms**  Health  OR  **Keywords**  Health OR Social Care | **Mesh terms**  Carbon Footprint OR  Greenhouse Gases OR Carbon Dioxide  **Keywords**  Carbon Footprint* OR Greenhouse Gas Emission OR Carbon Emission OR CO2 emission OR Sustainability |
