# Supplementary material for: Environmental Impact of Digital Health Care Technologies in Reducing Carbon Footprints: Scoping Review
Source: J Med Internet Res. 2026 Oct 21;28:e88341. doi: 10.2196/88341 (PMC13717332; doi:10.2196/88341)
Supplement: Supplementary file 2 [file jmir-v28-e88341-s002.docx]

**Appendix 2**: Literature search strategy: MEDLINE via EBSCOhost

| # | Query | Results |
| --- | --- | --- |
| S31 | S17 AND S21 AND S29 | 1313 |
| S30 | S17 AND S21 AND S29 | Display |
| S29 | S22 OR S23 OR S24 OR S25 OR S26 OR S28 | Display |
| S28 | AB sustainability OR TI sustainability | Display |
| S27 | AB “CO2 emission*” OR “CO2 emission*” | Display |
| S26 | AB “carbon emission*” OR TI “carbon emission*” | Display |
| S25 | AB “greenhouse gas emission*” OR “greenhouse gas emission*” | Display |
| S24 | AB “carbon footprint*” OR TI “carbon footprint*” | Display |
| S23 | (MH “Greenhouse Gases”) OR (MH Carbon Dioxide”) | Display |
| S22 | (MH “Carbon Footprint”) | Display |
| S21 | S18 OR S19 OR S20 | Display |
| S20 | AB “social care” OR TI “social care” | Display |
| S19 | AB health* OR TI health* | Display |
| S18 | (MH “Health”) | Display |
| S17 | S1 OR S2 OR S3 OR S4 OR S5 OR S6 OR S7 OR S8 OR S9 OR S10 OR S11 OR S12 OR S13 OR S14 OR S15 OR S16 | Display |
| S16 | AB telehealth OR TI telehealth | Display |
| S15 | AB telemedicine OR TI telemedicine | Display |
| S14 | AB CDSS OR TI CDSS | Display |
| S13 | AB “Clinical Decision Support System*” OR “Clinical Decision Support System*” | Display |
| S12 | AB DHT OR TI DHT | Display |
| S11 | AB m-health OR | Display |
| S10 | AB e-health OR TI e-health | Display |
| S9 | AB “digital health” OR TI “digital health” | Display |
| S8 | AB ehr OR TI ehr | Display |
| S7 | AB emr OR TI emr | Display |
| S6 | AB “electronic medical record*” OR TI “electronic medical record*” | Display |
| S5 | AB “electronic health” OR TI “electronic health” | Display |
| S4 | (MH “Decision Support System”) | Display |
| S3 | (MH “Digital Health”) | Display |
| S2 | (MH “Electronic Health Records”) | Display |
| S1 | (MH “Telemedicine”) | Display |
