# Supplementary material for: Environmental Impact of Digital Health Care Technologies in Reducing Carbon Footprints: Scoping Review
Source: J Med Internet Res. 2026 Oct 21;28:e88341. doi: 10.2196/88341 (PMC13717332; doi:10.2196/88341)
Supplement: Supplementary file 3 [file jmir-v28-e88341-s003.docx]

**Appendix 3: Excluded studies**

|  | Author and year | Article title | Reason for exclusion |
| --- | --- | --- | --- |
| 1 | Abi et al. | Feasibility and uptake of a digital mental health intervention for depression among Lebanese and Syrian displaced people in Lebanon: a qualitative study. | Wrong outcome/No assessment of environmental impact |
| 2 | Alami et al. | To What Extent Can Digital Health Technologies Comply With the Principles of Responsible Innovation? Practice-and Policy-Oriented Research Insights Regarding an Organisational and Systemic Issue. | Wrong outcome/No assessment of environmental impact |
| 3 | Allwright and Abbott (2021) | Environmentally sustainable dermatology | Review article |
| 4 | Bhopal et al. (2023) | Balancing the health benefits and climate mortality costs of haemodialysis. | Opinion paper |
| 5 | Bilgin (2025) | Beyond being a rheumatologist: Environment, climate change, and carbon footprint – We need an action! | Letter to a journal |
| 6 | Bloomfield et al (2021) | Artificial Intelligence in the NHS: Climate and Emissions | Review article |
| 7 | Bobini et al (2025) | Towards a Green Health Technology Assessment: embedding Life Cycle Assessment for sustainable choices | In other languages |
| 8 | Bobini et al. (2025) | Verso un Green Health Technology Assessment : il ruolo del Life Cycle Assessment per scelte sanitarie più sostenibili | In other languages |
| 9 | Bond et al. (2009) | Tackling climate change close to home: mobile breast screening as a model | Wrong intervention/The intervention assessed is not a DHT |
| 10 | Braybrooke et al. (2025) | Estimating the Cost and Carbon Output of Musculoskeletal Primary Care Management Decisions: A Retrospective Analysis of Electronic Health Records. | Wrong intervention/The intervention assessed is not a DHT |
| 11 | Calyx et al. (2023) | Embedding telehealth for sustainable health services linking Oceania | Report |
| 12 | Collins et al. (2024) | Implementing a Clinical Decision Support Tool to Reduce Operating Room Anesthetic Fresh Gas Flow: A Resident-Led, Sustainability-Focused Quality Improvement Initiative. | Wrong outcome/No assessment of environmental impact |
| 13 | Colquhoun et al. (2025) | Environmental and patient safety outcomes of a health-system Green Anesthesia Initiative (GAIA): a retrospective observational cohort study. | Wrong intervention/The intervention assessed is not a DHT |
| 14 | Cripps and Scarbrough, (2025) | Making Digital Health "Solutions" Sustainable in Healthcare Systems: A Practitioner Perspective | Perspectives/opinion papers |
| 15 | Das and Chandra (2023) | A survey on artificial intelligence for reducing the climate footprint in healthcare | Review article |
| 16 | Díaz Jiménez et al. (2024) | Assessing the sustainable alignment of a sensor-based connected health system with SDGs: an evaluation model and case study | Wrong outcome/No assessment of environmental impact |
| 17 | Donald and Irukulla (2023) | Greenhouse Gas Emission Savings in Relation to Telemedicine and Associated Patient Benefits: A Systematic Review. | Review article |
| 18 | Duane et al. (2019) | Environmental sustainability and travel within the dental practice | Discussion paper |
| 19 | Duijvestein et al. (2024) | The United European Gastroenterology green paper-climate change and gastroenterology. | Review article |
| 20 | Ebner et al. (2025) | First steps towards greater climate protection and sustainability in a gynecologic clinic | In other language |
| 21 | Ellis et al. (2013) | Making a case for telehealth: measuring the carbon cost of health-related travel | Wrong intervention/The intervention assessed is not a DHT |
| 22 | Fanta and Pretorius, (2023) | Sociotechnical factors of sustainable digital health systems: A system dynamics model | Wrong outcome/No assessment of environmental impact |
| 23 | Feldman (2028) | An early model for value and sustainability in health information exchanges: Qualitative study | Wrong outcome/No assessment of environmental impact |
| 24 | Fragão-Marques and Ozben, (2023) | Digital transformation and sustainability in healthcare and clinical laboratories | Perspectives/opinion papers |
| 25 | Gandhi et al. (2023) | Impact of virtual continued medical education on carbon footprint and awareness of digital sobriety: A retrospective cross-sectional study among public health professionals in India | Wrong intervention/The intervention assessed is not a DHT |
| 26 | Ghosh e al. (2024) | CARE: carbon-aware computing for blockchain-enabled internet of medical things | Wrong outcome/No assessment of environmental impact |
| 27 | Grau et al. (2025) | Endoscopic En Bloc Versus Piecemeal Resection of Large Colonic Adenomas: carbon footprint post-hoc analysis of a randomized trial. | Wrong intervention/The intervention assessed is not a DHT |
| 28 | Hansen et al. (2023) | Project SPRUCE: Saving Our Planet by Reducing Carbon Emissions, a Pediatric Anesthesia Sustainability Quality Improvement Initiative | Wrong outcome/No assessment of environmental impact |
| 29 | Hassane et al. (2024) | To What Extent Can Digital Health Technologies Comply With the Principles of Responsible Innovation? Practice-and Policy-Oriented Research Insights Regarding an Organisational and Systemic Issue. | Wrong outcome/No assessment of environmental impact |
| 30 | Hantel et al. (2024) | Assessing the Environmental and Downstream Human Health Impacts of Decentralizing Cancer Care. | Conference abstract |
| 31 | Irwin et al. (2024) | The carbon footprint of health care delivery in Western Australia's public health system | Wrong intervention/The intervention assessed is not a DHT |
| 32 | Kraybill et al. (2025) | Reducing Greenhouse Gas Emissions and Modifying Nitrous Oxide Delivery at Stanford: Observational, Pilot Intervention Study. | Wrong intervention/The intervention assessed is not a DHT |
| 33 | Kumar et al. (2025) | Integrated Smart Footwear: Advanced Health Monitoring and Energy Harvesting | Wrong outcome/ not assessment of environmental impact |
| 34 | Li et al. (2022) | Low-Carbon Action in Full Swing: A Study on Satisfaction with Wise Medical Development | Wrong outcome/No assessment of environmental impact |
| 35 | Lokmic-Tomkins et al. (2023) | Designing digital health applications for climate change mitigation and adaptation | Perspectives/opinion papers |
| 36 | Lokmic-Tomkins et al. (2023) | Interconnectedness of digital health, climate change and respiratory care: navigating an environmentally sustainable path forward | Book chapter |
| 37 | Lokmic-Tomkins et al. (2022) | Assessing the carbon footprint of digital health interventions: a scoping review. | Review article |
| 38 | Miranda et al. (2023) | Telemonitoring in Portugal: where do we stand and which way forward? | Review article |
| 39 | Mitchell-Gillespie et al. (2020) | Sustainable support solutions for community-based rehabilitation workers in refugee camps: piloting telehealth acceptability and implementation | Wrong outcome/No assessment of environmental impact |
| 40 | Muindi et al. (2025) | Critical review of environmental impact evaluations of electronic healthcare devices: challenges and recommendations | Review article |
| 41 | Muli et al. (2024) | Video Consultations and Environmental Sustainability - Usability's Impact on Long-Term Use. | Wrong outcome/No assessment of environmental impact |
| 42 | Palozzi et al. (2020) | Enhancing the sustainable goal of access to healthcare: Findings from a literature review on telemedicine employment in rural areas | Review article |
| 43 | Paquette and Lin (2019) | Outpatient Telemedicine Program in Vascular Surgery Reduces Patient Travel Time, Cost, and Environmental Pollutant Emissions | Conference abstract |
| 44 | Pérez-Díaz et al. (2022) | Degree of satisfaction and social and environmental impact in patients included in a remote pacemaker monitoring program | In other languages |
| 45 | Ploux et al. (2025) | Bridging the Gap: Remote Evaluation and Programming of Cardiac Implantable Devices in Medically Underserved Areas. | Wrong study type/intervention |
| 46 | Pradhan et al. (2021) | Virtual phone clinics in orthopaedics: evaluation of clinical application and sustainability | Wrong outcome/No assessment of environmental impact |
| 47 | Purohit et al. (2021) | Does telemedicine reduce the carbon footprint of healthcare? A systematic review. | Review article |
| 48 | Putnis and Neilson, (2022) | Environmental sustainability and quality care: Not one without the other | Commentary |
| 49 | Radhakrishnan et al. (2016) | Unsustainable Home Telehealth: A Texas Qualitative Study | Wrong outcome/No assessment of environmental impact |
| 50 | Ramaswamy et al. (2022) | An Accessible Clinical Decision Support System to Curtail Anaesthetic Greenhouse Gases in a Large Health Network: Implementation Study. | Wrong intervention/The intervention assessed is not a DHT |
| 51 | Rasheed et al. (2021) | Decarbonising healthcare in low and middle-income countries: Potential pathways to net zero emissions | Report |
| 52 | Ravindrane et al. (2022) | The environmental impacts of telemedicine in place of face-to-face patient care: a systematic review. | Review article |
| 53 | Rivard et al. (2024) | Thematic analysis of tools for health innovators and organisation leaders to develop digital health solutions fit for climate change | Review article |
| 54 | Rodler et al. (2023) | The Impact of Telemedicine in Reducing the Carbon Footprint in Health Care: A Systematic Review and Cumulative Analysis of 68 Million Clinical Consultations. | Review article |
| 55 | Rothwell et al. (2023) | Virtual appointments-embracing the opportunity to reduce carbon emissions mustn't widen health inequalities. | Perspectives/opinion paper |
| 56 | Rudrapatna et al. (2025) | A Comparative Environmental Impact Analysis of Screening Tests for Colorectal Cancer. | Wrong intervention/The intervention assessed is not a DHT |
| 57 | Salerno et al. (2024) | Transportation-Related Carbon Footprint of Coronary Heart Disease Ambulatory Care in the United States | Wrong intervention |
| 58 | Sattler et al. (2025) | Environmental Sustainability in the Outpatient Clinic Setting | Review paper |
| 59 | Savoldelli et al. (2025) | Integrating Social Impact Indicators into Environmental Analysis: A Proposal for Televisit Assessment | Not a peer-reviewed publication |
| 60 | Shaw et al. (2021) | Achieving Spread, Scale Up and Sustainability of Video Consulting Services During the COVID-19 Pandemic? Findings From a Comparative Case Study of Policy Implementation in England, Wales, Scotland and Northern Ireland | Wrong outcome/No assessment of environmental impact |
| 61 | Smith et al. (2022) | We can't mitigate what we don't monitor: using informatics to measure and improve healthcare systems' climate impact and environmental footprint | Perspectives/opinion papers |
| 62 | Stevenin et al. (2024) | e-Health and environmental sustainability in vascular surgery. | Review article |
| 63 | Sun et al. (2023) | Healthcare Built Environment and Telemedicine Practice for Social and Environmental Sustainability | Wrong outcome/No assessment of environmental impact/qualitative paper |
| 64 | Toma and Rea | The friendly hospital initiative for children and the computerized tool for monitoring and sustainability | In other languages |
| 65 | Tsagkaris et al. (2021) | Using telemedicine for a lower carbon footprint in healthcare: A twofold tale of healing | Short communication/commentary |
| 66 | Uddin et al. (2025) | Sustainable and Smart Healthcare-Based IIoT Tools | Book chapter |
| 67 | Usher et al. (2024) | The potential of virtual healthcare technologies to reduce healthcare services' carbon footprint | Perspective/opinion paper |
| 68 | VanderZee et al. (2024) | Assessing the Carbon Footprint of Telemedicine: A Systematic Review. | Review article |
| 69 | Wootton et al. (2010) | Environmental aspects of health care in the Grampian NHS region and the place of telehealth. | Discussion paper |
| 70 | Zou et al. (2016) | Environment and air pollution like a gun and bullet for low-income countries: war for better health and wealth | Wrong intervention |
