# Supplementary material for: Environmental Impact of Digital Health Care Technologies in Reducing Carbon Footprints: Scoping Review
Source: J Med Internet Res. 2026 Oct 21;28:e88341. doi: 10.2196/88341 (PMC13717332; doi:10.2196/88341)
Supplement: Supplementary file 4 [file jmir-v28-e88341-s004.docx]

| **ID** | **Author, year of Publication and country** | **Type of digital technology used** | **Study setting** | **Study design** | **Participants** | **Environmental impact assessment methods** | **Carbon footprint change/Environmental impact** |
| --- | --- | --- | --- | --- | --- | --- | --- |
| 52 | Moore et al. (2024).  United Kingdom | Virtual appointment (Video and telephone) | Hospital (transplant follow-up clinic) | Retrospective observational design | Patients underwent transplant surgery (528 virtual consultations) | Carbon footprint assessment based on travel. | Total distance saved:  42,666 miles. Total CO₂ emissions avoided:  11,718 kilograms (kg) of CO₂ equivalent. |
| 53 | Miah et al. (2019).  United Kingdom | Teleconsultation (landline or mobile phone) | Hospital (Urology clinic) | Prospective study | 409 Patients with urological conditions | Carbon footprint assessment based on travel. Carbon offsetting via tree equivalence | Total distance saved: 4623 travel miles. The estimated avoided carbon footprint -  0.35–1.45 metric tonnes of CO2e. The number of trees that would need to be planted to offset carbon production would be 1.75–7.25 trees. |
| 69 | Smith et al. (2007).  Australia | Teleconsultation (Video conferencing) | Hospital (Paediatric Burn service). | Retrospective study. | Paediatric patients with burn injuries (1000 consultations). | Travel distance saved. | The median travel distance of patients seen by telehealth  was 600 km (interquartile range 300–1225). In six years, the estimated travel savings  were about 1.4 million km. |
| 84 | Filfilan et al. (2021).  France | Teleconsultation (video connection) | Two  Academic Urology Departments | Prospective study | 80 patients attending the Urology Department | CARBON FOOTPRINT assessment based on energy consumption and travel. | CO2e emissions avoided due to the reduction in travel were 1.1 tonnes during the one-month period. Teleconsultation - 1.1 kg CO2e emissions. In-person consultations - 0.5 kg of CO2e. The total reduction in GHGs is 1141 kg CO2e, representing a 99% decrease in emissions. |
| 26 | Turley et al (2011).  USA | Electronic health records | Hospital and medical offices (Kaiser Permanente's Health Connect). | Retrospective analysis | Kaiser Permanente’s Health Connect registrants. | Environmental impact based on an Eco-Health footprint Model. Calculation of acres of carbon sequestration equivalents. | Annual environmental impact includes,  1. Greenhouse gases overall - 9200 CO₂e/year, required carbon sequestration equivalent - 3600 acres.  2. Paper use: 1372 tons, required carbon sequestration equivalent: 5550 acres.  3. Energy use-gasoline used for transportation 3.3- 10 million gallons, required carbon sequestration equivalent – 6400-19200 acres.  4. Energy use- personal computer (27300 MWh) and data centres (62000 MWh) - 89300 MWh, required carbon sequestration equivalent: 13300 acres.  5. Waste – X-ray plastic (68 tons) and personal computer plastic (135 tons)-203 tons, required carbon sequestration equivalent: 118 acres.  6. Overall toxic chemicals: 13 tons  7. Water use: 71.5 million gallons. |
| 64 | Masino et al. (2010)  Canada | Teleconsultation (video conferencing) | Academic health centres in hospitals. | Retrospective analysis. | Clinic registrants-840 consultations. | Carbon footprint assessment based on energy consumption and travel. | Reduced travel distances - total of 757,234 km ( GHG emissions avoidance of 185 metric tons of CO_2_eq). Avoided other air pollutant emissions: approximately 360,444 g. GHG emissions due to energy  Consumption by videoconference units: 42 kg of CO_2_eq. |
| 54 | King et al. (2023)  United Kingdom | Virtual consultation  (video and telephone) | General gastroenterology outpatient clinics at NHS Trusts. | Retrospective, observational, multisite,  cross-sectional study. | Patients attending the general  gastroenterology clinics. | Carbon footprint assessment based on travel and use of infrastructure. | In Group 1, emissions associated with patient travel were 1,165.29 kg CO2e, averaging 1.54kg/consultation. Carbon emissions for Group 2, which included personal transport and estimated emissions from phone calls, were 5.37kg CO2e. An average of 0.005kg per consultation.  Overall reduction of 1,159.92 kg CO2e (99.37%; p = 0.0001). |
| 55 | Bhavanasi &Kulkarni et a. (2024).  United Kingdom | Virtual clinic (foot and ankle clinic (Telephone)  Comparator: Face-to-face interview | Setting: NHS outpatient clinic | Retrospective analysis based on EHRs | 175 NHS outpatients | Carbon footprint assessment based on energy consumption, telephone usage, PPE, usage of water and staff and patients' commute | Performing an hour-long virtual consultation created  0.434kgCO2e per consultation compared to 5.264 kg CO2e for face-to-face. Patients travelled an average of 5.94 miles to attend the foot and ankle clinic, resulting in 3.33 kg CO2e emissions per round trip. Elimination of an estimated 1,040.4 miles of patient travel, equivalent to 291.31 kg CO2e over 4 months. In total, by avoiding face-to-face clinics, 63.22 kg CO2e of emissions were saved. |
| 85 | Oppelaar et al. (2024).  Netherland | Remote monitoring (spirometry tests) and teleconsultations (telephone or video) | Cystic fibrosis centres | Retrospective multicentre observational study | 81 people with cystic fibrosis | Carbon footprint assessment based on travel distance. Calculating carbon offsetting via tree equivalence. | The cumulative amount of GHG emissions was 41.7% lower in the first COVID-19 year and 22.7% lower in the second COVID-19 year compared to the pre-COVID-19 year. The total reduction in GHG emissions over the two COVID-19 years was 5,450.3 kgCO_2_e, equivalent to approximately 248 adult trees. |
| 27 | Danesh et al (2025).  USA | Tele-dentistry (Zoom). | Hospital (children) | Retrospective cross-sectional study | 101 children | Carbon emissions based on travel. | Tele-dentistry saved 13,822 patient miles of round-trip travel distance, with  a mean of 136.9 miles saved. Tele-dentistry visits resulted in 552.9 fewer gallons of gasoline consumed and  4,875.4 fewer kilograms of CO2 emissions from gasoline consumption compared with a hypothetical in-person office visit. |
| 28 | Kellerman (2025).  USA | Telemedicine visit (Video or telephone) | Tertiary Rhinology Centre | Retrospective analysis | Patients presenting for telemedicine visits (n=354). | Carbon emissions based on travel. | Approximately 64,977.00 miles and round-trip travel was saved, resulting in savings of an estimated  64,977.00 miles of driving, providing a reduction of 26,705.55 kg in CO2 emissions. |
| 29 | Martinez et al. (2024).  USA | Telemedicine (Video call). | Academic Tertiary Care Centre | Retrospective analysis | 12 patients with uncomplicated cholelithiasis | Carbon footprint assessment based on LCA | Twelve TPT patients’ virtual consultation was compared to thirty-one patients who  underwent elective cholecystectomy following an in-person preoperative visits over a 12-month period. TPT resulted in a 51% decrease in perioperative GHG emissions compared to patients who underwent in-person evaluation (60.2–60.9 kgCO2-eq vs. 123.4–123.5 kgCO2-eq, p=0.0271). |
| 86 | Li et al. (2025).  China | Telemedicine (online telemedicine platform/video) | Hospital | Retrospective analysis | Patients attended outpatient clinics | LCA based on the economic input-output model (travel and energy consumption). | Telemedicine could reduce GHG emissions by 36 tCO2e. The GHG emissions per telemedicine session were only 19.14 kgCO2e. Replacing in-person treatments with telemedicine  can lead to an average reduction of 85.51 kgCO2e emissions.  Transportation accounted for an average of 98.5% of GHG emissions during the patient treatment stage, making it the primary contributor to outpatient GHG emissions. |
| 76 | Desenzi Ciaralo et al. (2025).  Brazil | Telemedicine/Tele-trachea Consultation (Online) | Public hospital: Academic | Cross-sectional study based on EHRs | 1767 telemedicine visits with 680 patients | CO2 emission based on travel. | The estimated distance saved in kilometres  was 2.219.544,3 km (median savings per patient [IQR] 542,88 km [190,36-2.672,6]) corresponding to a 353.097,55 kg of CO2 in emissions savings (median CO2 emissions per patient [IQR] 102,56 kg  [36,56-496,96]. |
| 87 | Ozen et al. (2024).  Turkey | Telemedicine  (technology not stated) | Public hospital: Academic | Retrospective analysis based on patient records. | 1767 telemedicine visits with 680 patients | Carbon footprint assessment based on travel. | Telemedicine for cardiovascular disease care could have reduced patient travel by an estimated 656,258 km if the initial consultation had been conducted in person and all subsequent follow-up visits had been delivered via telemedicine instead of face-to-face appointments. The travel-associated CARBON FOOTPRINT saving was 30%. |
| 65 | Mainer-Pearson et al. (2025).  Canada | Telehealthcare appointment (telephone and online) | Hospital Emergency Department | Secondary data analysis | Patients attending the Emergency Department | Carbon footprint assessment based on travel. | Rural CO2-equivalent emissions per visit (kg) 13.92, Urban 2.89.  n, avoiding  in-person care diverts up to 13 kg of CO2 per medical visit, depending on the distance and frequency of travel to appointments. |
| 88 | Meryk et al. (2025)  Austria | Telemedicine (Video consultation) | University Medical Unit: Paediatric Oncology | Single-centre cohort study | 22 paediatric patients with acute leukaemia | CO2 emissions based on travel. | The implementation of the IHCN resulted in savings of 70,158 km, 950 hours of travel, and 12,277 kg of CO2 Emissions. |
| 66 | Galts et al. (2025).  Canada | Telemedicine (video or telephone) | Academic Gastroenterology Clinic | Design: Cross-sectional analysis. | Clinic attendees (975 appointments). | CO2 emission based on travel.  Carbon offsetting via tree equivalence  The number of litres of gasoline burned. | The mean return distance travelled per appointment is 57.3 km, which translates to 14.9 kg CO2 per patient visit. An average day at our clinic was equal to burning 146.6 L of gasoline or the annual carbon capture of 15.5 trees. |
| 56 | Dorrian et al. (2009).  United Kingdom | Telemedicine (tele-endoscopy images and video conferencing) | Tele-endoscopy remote clinic. | Feasibility study | 42 patients | CO2 emission based on travel. | A total of 42 journeys were avoided, each journey saving 123 kg CO2 per person. |
| 57 | Lewis et al. (2009).  United Kingdom | Telemedicine (Video conferencing) | Setting: Hospital/cancer care | Survey | 60 patients. | CO2 emission based on travel.  Carbon offsetting via tree equivalence. | During October  In 2007, a total of 20,800 km of car travel was avoided, equivalent to 2590 kg of CO2 emissions. 48 trees  would take a year to absorb that quantity of CO2. |
| 89 | DiGiacomo & Håkansson (2011).  Croatia | eHealth application /electronic referral system | Primary care | Secondary data analysis based | Participants: potential citizens | carbon footprint assessment based on LCA | The two services in the e-Health system account for about 330 metric tons of CO2e emissions per year. PCs and networks account for over 90%, and the data centre accounts for the rest. Given that patients reduce their travel by an average of almost 3 visits per year, the potential reduction in travel-related CO2e emissions is approximately 7 kg per patient/year. This is a result of a reduction of up to 15,000 metric tons of CO2e, provided 50% of all travel can be avoided. |
| 30 | Wong et al. (2025)  USA | Telehealth | Urology academic unit | Retrospective analysis/electronic health record query | 6444 patients | CO2 emission based on travel. | Avoided CO2 emissions are 153.36 metric tons |
| 12 | Markovic et al. (2025).  Serbia | Telemedicine consultation (video). | Hospital. | Retrospective analysis | 107 patients with  Crohn's disease and ulcerative colitis. | Carbon footprint assessment based on travel. | The average distance saved per patient was 168.28 km, totalling 18,006 km saved.  The reduction in CARBON FOOTPRINT was 326 tonnes, which is significant  Considering that the annual average carbon footprint is 4.65 tons |
| 31 | Dullet et al. (2017).  USA | Telemedicine (video technology). | University  Telemedicine Program | Retrospective analysis | 38,051  consultations. | Reduction in pollutants and GHG emissions based on travel. | The use of telemedicine  resulted in a travel-related emissions reduction of 1969 metric tons of CO2, 50 metric tons of CO, 3.7 metric tons of N2O, and 5.5 metric tons of volatile organic compounds |
| 80 | Vidal-Alaball et al. (2019)  Spain | Telemedicine (Remote review through EHRs and telephone) | Primary health centres, tertiary referral hospitals. | Retrospective analysis. | 12,322clinic referrals. | Atmospheric pollutants and GHGs based on travel. | The distance saved was 192,682 km, with a total travel time saving of 3779 h and a total fuel reduction of 11,754 L. This represents an average reduction of 3248.3 g of CO2, 4.05 g of CO, 4.86 g of N2O and 3.2 g of sulphur dioxide. |
| 58 | Connor et al. (2019).  United Kingdom | virtual ureteric colic clinic  (telephone consultation) | Tertiary care clinic | Prospective study. | Patients with uncomplicated acute ureteric colic | Carbon footprint assessment based on travel.  Carbon offsetting via tree equivalence. | The mean (IQR) distance travelled by each patient was 4.3 miles. The carbon footprint attributable to travel avoidance was equated to 0.70 -2.93  metric tonnes of CO2e. Offsetting this carbon footprint would require planting 14.7 trees. |
| 59 | Sellars et al. (2020).  United Kingdom | Video consultation | Hospital | Prospective observation study. | 50 patients with colorectal cancer | Carbon footprint assessment based on travel. | The total number of travel miles saved by Near Me appointments was 6685. This represents an estimated time saving of 148 h and carbon emissions saving of 4659 lb CO2e. |
| 32 | Mojdehbakhsh et al. (2021).  USA. | Telemedicine | University Cancer Centre  clinic | Quality improvement study with a survey. | 408 total outpatient encounters | CO_2_ emissions based on travel. | Travel distance was 15,511.8 miles for 192 patients.  6.25 metric tons of CO2 emissions from travel were prevented. |
| 90 | Croghan et al (2021).  Ireland | Virtual Urology clinics. (Telephone) | Urology clinic. | Retrospective analysis | 1,016 consultations | CO_2_ emissions based on travel.  Carbon offsetting via tree equivalence. | An overall reduction in round-trip distance travelled by patients of 31,038 miles. Mean saving of  93.8 miles per rural-dwelling patient. Estimated reduction of 6.07 tonnes of CO2 emissions, based on the predicted carbon footprint of car-travelling patients alone.  Absorbing this amount of CO₂ emissions in one year would require 434 mature (10-year-old) evergreen trees. |
| 70 | Barakat-Johnson et al. (2022).  Australia | Virtual wound care | Community virtual wound care centre | Mixed-method study | Fifty-one patients with  chronic wounds | Carbon emissions and travel. Carbon offsetting via tree equivalence. | For remote participants, CO2 emissions were reduced by 250 kg per round trip. Around 30 to 850 trees per patient would be required to be planted to offset carbon emissions by participants accessing the wound service. |
| 33 | Roy et al. (2023).  USA | Telemedicine (telephone and video) | University GI clinic | Retrospective cohort study | Patients who underwent telemedicine visits | Carbon footprint assessment based on travel. | Total distance of 8732 miles that patients would have travelled for in-person visits (including return trips). The average travel distance calculated (a composite of the drive to and from the facility) was 78.67 miles. A total of 393.3 gallons of gasoline would have been used for travel. Per EPA calculators, a total of 3.5 metric tons of GHG emissions were prevented by performing tele visits in lieu of in-person visits due to the avoidance of fuel use. This averages to 31.5 kg of GHG emissions and 3.54 gallons of gasoline saved per patient. |
| 77 | Ciaralo et al. (2024).  Brazil | Telemedicine consultation (online video) | Academic hospital. | Retrospective analysis based on EHRs. | Patients with central airway diseases (n= 1153) | CO_2_ emission based on travel. | The travel savings were 1,224,108.54 km, corresponding to a 250.14-ton reduction in CO2 emissions. |
| 73 | Savoldelli et al (2024).  Italy | Telemedicine (Video calls) | Setting: Hospital. | LCA design | Chronic heart failure patients. | LCA based on energy consumption, travel and medical waste. | Tele visit  reduces emissions from 9.77 kgCO2e to 0.41 kgCO2e. Transport and internet data use are key inputs for in-person (i.e., 98%) and telemedicine visits (i.e., 72%), respectively. |
| 78 | Rani et al. (2024).  India | Teleopthalmology (Video call) | Primary and Tertiary eye care unit. | Prospective study with a survey | 324 (primary: 173; tertiary: 151) patients | CO_2_ emission based on travel. | The primary rural network saved 2.89 kg CO2/person and 80 km/person. The tertiary urban network saved 176.6 kg CO2/  person and 1666 km/person. |
| 35 | Tarpani & Gallego-Schmid (2024).  United Kingdom | Digital health and well-being service | Elderly living scheme. | LCA study | Residents of three elderly living schemes | LCA for living hub, door entry, video desk phone, handset, pendant and base unit. | The digital service’s estimated global warming potential ranges from 718 to 1,509 kg CO2eq/resident.  Electricity consumption was found to be the main contributor to most environmental impacts. |
| 82 | Muschol et al. (2022).  Germany | Digital health app + video  consultations | University hospital. | Prospective randomised controlled trial | 52 patients | Emission of GHGs based on travel and assessment of other pollutants. | Emissions of 11.248  kg of GHGs, 0.070 kg of CO, 0.011 kg of volatile hydrocarbons, 0.028 kg of N2O, and 0.0004  kg of particulates could be saved per patient through avoided travel.  As a result of the video consultations,  emissions of 292.448 kg of GHGs could be avoided.  With a total of 384 patients who would not have to travel to the clinic each year due to video consultations, a total of 4009.88 kg of greenhouse gases, 24.80 kg of CO, 3.96 kg of volatile hydrocarbons, 10.02 kg of N2O, and 0.16 kg of particulates could be avoided. |
| 71 | Schulz et al. (2020).  Australia | Telehealth consultation (telephone). | Hospital | Retrospective quality audit. | 58 patients with hepatitis C. | Carbon emissions based on travel. | he median travel distance avoided per telehealth consultation was 634 km (mean: 626 km). Patients attended a median of three consultations (mean: 3.06) during their treatment course, with a median of two consultations (mean: 2.4) delivered via telehealth. Over the course of one year, an estimated 86,720 km were saved by seeing patients via telehealth. This will have reduced the carbon output by a total of 22.37 tons. |
| 74 | Materazzo et al. (2022).  Italy | Intervention: Telehealth (telephone)  Comparator: not reported. | Hospital | Retrospective cohort study | Patients in the postoperative  follow-up after breast conserving procedure (69 pre-COVID-19 and 69 COVID-19 groups). | Travel | A reduction of 4312.38  km in travel to the hospital was found in the COVID-19 group. |
| 81 | Maria et al. (2022).  Spain | Intervention:  Telemedicine. | Hospital | Secondary data analysis. | Inhabitant and municipality to specialist | Carbon footprint assessment based on travel. | In total, 79 metric tons of carbon dioxide, 74.52 kg of methane and 487.27 kg of nitrous oxide could be avoided per year. For each specialist consultation carried out by the RHCs, emissions of 3.678 kg of CO2 equivalent (3.652 kg CO2, 3.45 g of methane and 22.6 g of nitrous oxide) would be avoided.  n total, the completion of these 21,548 visits from the RHCs represents a mileage saving of 447,279km and 38,019 L of fuel savings. |
| 34 | Robinson et al. (2017).  USA | Telemedicine (telephone pre-screening). | University-based tertiary referral centre. | Retrospective analysis based on data from records and observations. | 161 patients waiting for surgery | CO_2_ emission based on travel. | The telephone  The pre-screening appointment saved 106,070 miles of transportation. A total reduction of CO2 is 43,595 kg of CO2 emissions. |
| 35 | Wang et al. (2021).  USA | Telehealth screening (telephone) | Hospital | Retrospective cross-sectional analysis based on EHRs. | 298 adults ASA I-III patients undergoing elective spinal surgery | CF assessment based on preoperative testing, clinic visits, and patient commuting. | GHG emissions were reduced by 8.09 kg CO2e (9.6%) per patient, from 84.52 kg CO2e preintervention to 76.43 kg CO2e post-intervention (p = 0.019). The largest reduction, 14.71 kg CO2e, stemmed from fewer in-person clinic evaluations. An additional reduction of 18.07 kg CO2e per patient would have been possible if clinic performance were optimized (only necessary testing performed), resulting in a total reduction of  26.16 kg CO2e (31%). |
| 36 | Dacones et al. (2021).  USA | Telehealth: virtual appointments (audio-video or audio-only) | Outpatient care | Retrospective analysis. | 15.6 million outpatient visits during 2015-2020 | CF assessment based on travel. | Outpatient visits increased by 3.2% annually, to 2.7 million. Telehealth visits increased by an average of 53.2% annually, while in-person visits saw modest gains of 1.5% annually until by 2020, they declined by 46.2%. Transportation GHG emissions rose from 18.5 to 19.6 (in 2019) before declining to 10.5 kt CO2-eq in 2020. Ambulatory visit carbon intensity monotonically declined from 8 to 4 kg CO2-eq per visit. |
| 46 | Jiang et al. (2021).  USA | Telehealth (Audio-visual or telephone). | Veterans Affairs (VA) health care system. | Survey/single-centre study. | 100 veterans | CO_2_ emission based on travel. | A cumulative 86,470 miles of travel (mean travel savings 154.4 miles per patient encounter. Transitioning to tele-oncology was responsible for an estimated carbon  footprint reduction of 35.5 metric tons of CO2, approximately 106 metric tons of CO2 on an annualised basis  in savings. |
| 72 | Andrew et al. (2020).  Australia | Telehealth services (video calls) | Hospital. | Mixed-method case study. | 263 clinical reviews. | Carbon footprint assessment based on travel. | Total saved travel distance was 203,202 kilometres (equivalent to five trips around the circumference of the world), 2771 hours (115 days) in car travel time approximately/ reduction in greenhouse gas emissions of 51 tonnes CO2 equivalents. |
| 61 | Bartlett & Keir (2022).  United Kingdom | Virtual consultation (Telephone and video) | Geriatric Medicine  Clinic. | Record analysis | Clinic attendees/staff | Carbon footprint assessment based on patient travel, staff travel, PPE, water, waste, telecommunications and heating/lighting of the clinic facilities. | The carbon footprint of a face-to-face clinic consultation is 4.82 kgCO2e, most of which is patient travel, followed by staff travel and use of PPE. The footprint of a virtual consultation is 0.99 kgCO2e, with most of it attributable to staff travel, followed by data use. |
| 37 | Whetten et al. (2019).  USA | Teleconsultation | Academic health centre and rural hospital. | Record analysis | 2020 consultations. | Carbon footprint assessment based on travel | Emission reduction totalled 618,772 kg of CO2es (CO2e) (618.77 metric  tons) or 0.306 metric tons of CO2e per patient. Expanding the program resulted in  potential reductions of 4,307 (IQR 3,386–5,274) and 213,279 (IQR 169,320–263,570) metric tons of CO2e. |
| 38 | Thota et al. (2020).  USA | Telehealth (video call). | Hospital cancer clinic | Retrospective analysis. | 119 patients with 1,025 encounters | CO_2_ emission based on travel. | Reduced carbon emissions by approximately 150,000 kg for the 119 patients over the 4-year period.  Per patient, this results in a reduction of 1,334 kg of carbon emissions. |
| 39 | Sillcox et al. (2023).  USA | Telemedicine (video conferencing) | General surgery subspecialty clinic. | Retrospective analysis. | 145 patients | LCA | For in-person visits, 145 patients' travel distances were recorded with a median [IQR] travel distance of  29.5 [13.7, 85.1] miles, resulting in 38.22–39.61 CO2es (kgCO2-eq) emitted. For telemedicine visits, the  mean (SD) visit time was 40.6 (17.1) min. Telemedicine GHG emissions ranged from 2.26 to 2.99 kgCO2-eq depending on the device used. An in-person visit resulted in 25 times higher GHG emissions than a telemedicine visit (p<0.001). |
| 40 | Iaccarino et al (2022).  USA | Telerehabilitation (video visit). | Setting: Hospital rehabilitation unit. | Retrospective chart analysis and patients’ satisfaction survey | Patients/ 54 synchronised video visits. | GHG emission based on travel.  Carbon offsetting via tree equivalence. Home electricity uses as a contextual equivalence. | In aggregate, SVV saved an accumulated 13,618 miles.  The reduction in CO2 emissions is equivalent to the carbon sequestered by 181 tree seedlings grown for  10 years or one family of four home electricity use for 12 months. |
| 41 | Thiel et al. (2023).  USA | Virtual clinic  (telephone and video) | Setting: Academic medical centre | LCA with retrospective analysis | Clinic attendees. | Carbon footprint assessment through LCA.  CO2 sequestered by acres of US forest in one year. Home electricity use serves as a contextual equivalence. | During this period, Stanford Health Care reported an estimated 36% reduction in GHG emissions associated with clinic visits, declining from around 40,600 metric tons of CO₂e to 25,900 metric tons. In 2021, average in-person visit emitted an estimated 20 kg CO2e, while a phone-based virtual visit emitted only 0.02 kg CO2e and a video visit  emitted 0.04 kg CO2e on average. Electricity used on computers and phones during virtual visits in 2021 resulted in 29,000 kg  CO2e, equivalent to burning 3252 gallons of gasoline or the energy usage of 3.6 homes for one year. SHC’s virtual care system reduced their 2021 GHG emissions by nearly 17,000 metric  tons as compared to treating those same patients in  person. This is the equivalent of over 2100 homes energy use for a  year or the CO2 sequestered by nearly 20,000 acres of US forest in one year. |
| 42 | Moore et al. (2024).  USA | Electronic consultation (eConsult serve). | Setting: Academic Medical Centre | Retrospective observation study | Clinic attendees/15,499 eConsultations | Carbon footprint assessment based on travel. | In-state travel distance saved was 310,858 miles, travel time saved was 5,491 h, with an associated fuel reduction of 13,575 gallons and $56,893 savings. This  reduced GHH emissions by 128 metric tons of CO2, 0.022 tons of N2O, 0.005 tons of methane,  and 0.001 tons of nitrous oxide. Out of state travel distance saved was 188,346 miles with 2,842 h reduced travel time, and associated fuel reduction of 8,225 gallons and of 34,118. Reduced GHG emissions were equivalent to 77 metric  tons of CO2, 0.0132 tons of N2O, 0.0033  tons of methane, and 0.0007 tons of nitrous oxide. |
| 62 | Blenkinsop et al. (2021).  United Kingdom | Remote teleclinics (video and telephone) | Setting: Hospital | Retrospective analysis based on medical records. | 1200 epilepsy patients | Carbon footprint assessment based on travel and energy consumption | The saving of ~224 000 km of travel with likely avoided emissions in the range of 35 000–40 000 kg CO2e over a six and half month period. Emissions arising directly from remote delivery were calculated to be <200 kg CO2e (~0.5% of those for travel), representing a significant net reduction of GHG emission. |
| 75 | Papavero et al (2023).  Italy | Telemedicine | Hospital | Quality improvement study | Patients and carers /2,091 tele visits. | CO_2_ emission based on travel. | The impact on the environment was expressed by a total amount of CO2 emission savings of -16 tons. A single tele-medicine visit can contribute with a median of carbon emission savings equal to - 13 kg (IQR: 6–24). |
| 91 | Schmitz-Grosz et al. (2023).  Swisterland | Telemedicine | Primary care | Retrospective study | Patients/433 890 telemedical consultations | Carbon footprint assessment based on a comprehensive footprint approach | 433 890 telemedical consultations overall helped save 1 800391km in travel. On average, 1 telemedical  consultation saved 4.15 km of individual transport and consumed 0.15 kWh. After subtracting the CO2eq emissions caused by the telemedical center, the data reveal savings of 247.1 net tons of CO2eq emissions in total and of 0.57 kg CO2eq per telemedical consultation. |
| 63 | Lathan et al. (2024).  United Kingdom | Remote monitoring (Photograph). | Tertiary vascular centre  . | Pilot cohort study | Patients who underwent a lower limb vascular procedure. | LCA: a hybrid life-cycle assessment approach.  Carbon offsetting via tree equivalence. | The median return distance for patient travel was 42.5 (7.2–58.7) km. Median reduction in emissions using remote follow-up was 41.2 (24.5–80.3) kgCO2e per  patient (P < 0.001). The carbon offsetting value of remote follow-up is planting  one tree for every 6.9 patients. Total carbon footprint of face-to-face  follow-up was 2,895.3 kgCO2e, compared with 1,301.3 kgCO2e when using a remote-first approach (P < 0.001). Carbon emissions due to participants  without SSI were 700.2 kgCO2e by the clinical method and 28.8 kgCO2e from the remote follow-up. |
| 43 | Sharma et al. (2023).  USA | Ambulatory telehealth (audio-only and audio-video). | University healthcare system- Ambulatory care. | Retrospective analysis | Patients who seek ambulatory telehealth services (n=3,043,369). | GHG emissions based on travel. | The total round-trip distance saved from encounters was 53,664,391 miles.  The use of telehealth for ambulatory services during this time eliminated 21465.8 metric tons of CO2, 14.1 metric tons of total hydrocarbons, 212.3 metric tons of exhaust CO, and 9.3 metric tons of exhaust N2O emissions. |
| 92 | Dieperink et al. (2023).  Denmark | Video consultation technology. | Hospital. | Mixed-method study | 85 patients with gastrointestinal cancer. | Travel and CO2 emission. | All patients avoided coming to the hospital for at least  one consultation. For the 75 (89%) patients who primarily used their own car for transportation, 38 (46%) saved an hour or more of travel time and avoided travelling a median distance of 120 km (2–450 km) for each video consultation. The total distance saved was 12,877 km,  equivalent to 7018 lb of CO2. This CO2 emission corresponds to 387,226 smartphone charges. |
| 93 | Oliveira et al. ( 2013).  Portugal | Teleconsultation (video conferencing) | Hospital | Survey | 100 teleconsultation patients  and 100 face-to-face outpatients. | Carbon footprint assessment based on travel | The total direct GHG emissions is 0.19469 kg CO2e/km. In Portugal, this would be 0.18106 kg CO2e/km (indirect emissions are not affected by this adjustment). Total GHG emissions are approximately 510 tCO2e. |
| 44 | Cockrell et al. (2022).  USA | Telehealth (telephone or video conferencing). | Paediatric surgical care facility | Retrospective cohort study | Paediatric patients (60,773 in-person and 10,626 telehealth encounters) | Travel and CO2 emission.  Carbon sequestration equivalent-land in acres. Number of barrels of gasoline burned | Telehealth resulted in 887,006 patient-miles saved.  CO2 emissions prevented were equivalent to the CO2 emissions from burning more than 720 barrels of oil or the CO2 sequestered by 383 acres of US forest in one year. |
| 45 | Gunn et al. (2024)  USA | Telehealth visit | Hospital | Retrospective analysis | 9942 adult patients with telehealth visits within the Division of Cardiology / 14,828 cardiology telehealth  Visits. | GHG emissions based on travel.  Carbon offsetting via tree equivalence. | Over 45 months period, 14,828 telehealth visits among 9942 patients saved 484,152 kg of CO2 5225 kg of CO, 243,491 g of NO  X , and 9091 g of PM  2.52, with the total carbon saved equivalent to planting 9070 tree saplings over ten years. |
| 67 | Heffernan et al. (2024).  Canada | Virtual visit (not technology stated) | Children's Hospital. | Modelling cohort study design. | Paediatric patients with obstructive sleep apnoea or otitis media with effusion. | Carbon footprint assessment based on travel through the modelling approach. | Potential emissions savings of 1,2, or 3 virtual visits in the obstructive sleep apnea care pathway  were 19.9%, 39.9%, and59.8% respectively.  Potential emissions savings for integrating 1, 2, or 3 virtual visits into the otitis media with effusion care pathway were 16.6%, 33.2%, and 49.7%, respectively.  Integrating 3 virtual visits can save up to 2,156.8 kg CO2e per patient. |
| 62 | Sillcox et al. (2023).  USA | Telemedicine  (audiovisual conferencing) | Hospital clinic | Retrospective study | 51 in-person visits and 55 virtual visits. | CO_2_ emission based on travel | The first cohort accounted for a total of 25,310 patient car miles travelled, resulting in an estimated 10,225 kg of CO₂ emissions. The second cohort accounted for 25,310 patient car miles travelled and an estimated 10,225 kg of CO₂ emissions. |
| 39 | Penaskovic et al. (2022).  USA | Virtual care (telephone and video) | Setting: University psychiatric clinic | Retrospective analysis. | 3975 clinic attendees, 47,582 outpatient behavioural health visits. | CO2 emission based on travel.  GHG emissions in relation to a passenger vehicle driven for one year. | After accounting for emissions generated by patient technology use during telehealth visits, the estimated net reduction in GHG emissions was 867,011 kg CO₂. This reduction is equivalent to the annual GHG emissions produced by approximately 189 passenger vehicles. |
| 83 | Arndt et al. (2023).  German | Video consultation. | University hospital outpatient clinic. | Survey | 51 consultations | CO_2_ emission based on travel. | A video consultation resulted in a 97% reduction in GHG emissions compared to a face-to-face presentation. Implementation of video consultations reduces GHG emissions by over 0.5 tons of CO2e for respondents compared with patients travelling by car. |
| 79 | Kwon et al. (2024).  India | Electronic medical records | Hospital: eye care facility | Retrospective analysis | Patients | Carbon footprint assessment based on LCA. | The EMR system is associated with substantially higher GHG emissions than the paper-based medical record system, generating 195,000 kg CO₂e annually (0.361 kg CO₂e per patient visit) compared with 20,800 kg CO₂e annually (0.037 kg CO₂e per patient visit) for the paper-based system. |
| 94 | Holmner et al. (2014).  Sweden | Telemedicine/Tele-rehabilitation (video conferencing). | Hospital - rehabilitation unit and plastic surgery unit. | Secondary data analysis/Record analysis | Clinic attendees | LCA | Telemedicine consultations reduced carbon emissions by approximately 40–70-fold compared with conventional face-to-face visits.  Based on the upper- and lower-bound scenarios, a one-hour telemedicine appointment was estimated to generate 1.86 and 8.43 kgCO2e, respectively. |
| 49 | Cummins et al (2024).  USA | Telemedicine (video conferencing) | National telehealth platform/Doxy.me | Retrospective observational study. | Telemedicine session attendees/8,000,000 telemedicine sessions. | CO_2_ emission based on travel. | The mean CO2 savings per telemedicine session is 54,077 (SD 79,362) g, and the median was 19,812 (IQR 8322-58,772) g. The mean CO2 savings per minute of telemedicine care was 2896 (SD 6258) g, and the median is 723 (IQR 239-2597)g.  Energy: CO2 emissions equivalent of telemedicine energy use Mean-8.07 (4.68) Median - 8.59 (3.39-12.09)  Minimum to maximum - 1.17 to 28.0. |
| 68 | Lambert et al. (2023).  Canada | Virtual visit/video conference | Cancer care system | Retrospective analysis. | Patients with cancer. | CO_2_ emission based on travel. | The estimated travel  distance saved varied from 420,000 to 750,000 km per month. Estimated CO2 emissions prevented varied from 87 to 155 metric  tons per month. |
| 50 | Patel et al. (2023).  USA | Telemedicine/video conferencing. | Cancer institute. | A retrospective cross-sectional study. | A total of 49,329 telemedicine visits with 23,228 patients. | C0_2_ based on travel. consumed  Home electricity used. Home energy use.  Tree seedlings grown for 10 year and Acres of US forests in 1 year. | For patients who lived within a 60-minute driving distance of MCC, an estimated 1,032,775 round-trip miles were saved, corresponding to an estimated 424,471 kg of CO2 emission savings. Per-visit, mean (SD) savings of 48.1 (22.1) miles and 19.8 (19.4) CO2 kg emissions were reported. For patients whose driving distance to MCC exceeded 60 minutes, 6,677,002 round-trip miles were saved, corresponding to an estimated 2,744,248 kg of CO2 emissions. Per-visit, mean (SD) savings of 239.8 (84.2) miles and 98.6 (54.8) CO2 kg emissions were reported. |
| 51 | Grabski et al. (2024).  USA | Telemedicine visit | Outpatient Children's Hospital Centre. | A review of a prospective electronic medical record. | Children aged 0−18 years/ 20,845 paediatric telemedicine visits. | CO2 emission based on travel. | The travel distance  eliminated was 1,562,716 miles (round trip), representing a savings of 69,454 gallons of fuel, which translates to 618 metric tonnes of CO2 saved. |

References:

12. Marković S, Kralj D, Knežević Ivanovski T, Svorcan P. Telehealth-an environmentally friendly way to take care of patients with inflammatory bowel disease. Medicina (Kaunas). Feb 14, 2025;61(2):332. [doi: ] [Medline: 40005450]

26. Turley M, Porter C, Garrido T, et al. Use of electronic health records can improve the health care industry’s environmental footprint. Health Aff. May 2011;30(5):938-946. [doi: ]

27. Danesh DO, Luca J, Kalady SE, Townsend JA, Hammersmith KJ, Meyer BD. The impact of teledentistry on travel distance and carbon emissions at a children’s hospital. J Am Dent Assoc. Feb 2025;156(2):144-150. [doi: ] [Medline: 39692641]

28. Kellerman H, Mace JC, Detwiller KY, Geltzeiler M, Smith TL, Pandrangi VC. Travel costs and carbon savings associated with telemedicine in a tertiary care rhinology center. Int Forum Allergy Rhinol. Jul 2025;15(7):692-698. [doi: ] [Medline: 39918555]

29. Martinez KA, Haider A, Tarefder R, Khan Z, Meiklejohn DA, Zhu J. Piloting total perioperative telemedicine for elective cholecystectomies for symptomatic cholelithiasis. Surg Endosc. Mar 2025;39(3):1811-1816. [doi: ] [Medline: 39821454]

30. Wong V, Cohen J, Ingram A, et al. Patient-centered cost saving and positive environmental impact with the introduction of telehealth services at a single center. Urol Pract. Jan 2025;12(1):44-50. [doi: ] [Medline: 39356577]

31. Dullet NW, Geraghty EM, Kaufman T, et al. Impact of a university-based outpatient telemedicine program on time savings, travel costs, and environmental pollutants. Value Health. Apr 2017;20(4):542-546. [doi: ] [Medline: 28407995]

32. Mojdehbakhsh RP, Rose S, Peterson M, Rice L, Spencer R. A quality improvement pathway to rapidly increase telemedicine services in a gynecologic oncology clinic during the COVID-19 pandemic with patient satisfaction scores and environmental impact. Gynecol Oncol Rep. May 2021;36:100708. [doi: ] [Medline: 33521218]

33. Roy W, Hans B, Jannat RU, Reddy YK, Hadi Y, Gayam S. Tele-visits for GERD: “Ecofriendly, efficient and effective”. J Gastroenterol Hepatol. Jun 2023;38(6):905-909. [doi: ] [Medline: 36813701]

34. Robinson JD, Prochaska JD, Yngve DA. Pre-surgery evaluations by telephone decrease travel and cost for families of children with cerebral palsy. SAGE Open Med. 2017;5:2050312117720046. [doi: ] [Medline: 28839936]

35. Wang EY, Zafar JE, Lawrence CM, et al. Environmental emissions reduction of a preoperative evaluation center utilizing telehealth screening and standardized preoperative testing guidelines. Resour Conserv Recycl. Aug 2021;171:105652. [doi: ]

36. Dacones I, Cave C, Furie GL, Ogden CA, Slutzman JE. Patient transport greenhouse gas emissions from outpatient care at an integrated health care system in the Northwestern United States, 2015–2020. J Clim Chang Health. Aug 2021;3:100024. [doi: ]

37. Whetten J, Montoya J, Yonas H. Access to better health and clear skies: telemedicine and greenhouse gas reduction. Telemed J E Health. Oct 2019;25(10):960-965. [doi: ] [Medline: 30359184]

38. Thota R, Gill DM, Brant JL, Yeatman TJ, Haslem DS. Telehealth Is a sustainable population health strategy to lower costs and increase quality of health care in rural Utah. JCO Oncol Pract. Jul 2020;16(7):e557-e562. [doi: ] [Medline: 32463765]

39. Sillcox R, Gitonga B, Meiklejohn DA, et al. The environmental impact of surgical telemedicine: life cycle assessment of virtual vs. in-person preoperative evaluations for benign foregut disease. Surg Endosc. Jul 2023;37(7):5696-5702. [doi: ] [Medline: 37237107]

40. Iaccarino MA, Paganoni S, Tenforde A, et al. Environmental impact of telerehabilitation visits in an urban setting. J Clim Chang Health. Oct 2022;8:100150. [doi: ]

41. Thiel CL, Mehta N, Sejo CS, et al. Telemedicine and the environment: life cycle environmental emissions from in-person and virtual clinic visits. NPJ Digit Med. May 9, 2023;6(1):87. [doi: ] [Medline: 37160996]

42. Moore SL, Grim S, Kessler R, Luna MLD, Miller DE, Thomas JF. Reduction of environmental pollutants and travel burden through an academic medical center-based electronic consultation program. Telemed J E Health. Apr 2024;30(4):1020-1025. [doi: ] [Medline: 38064483]

43. Sharma S, Yellowlees PM, Gotthardt CJ, Luce MS, Avdalovic MV, Marcin JP. Environmental impact of ambulatory telehealth use by a statewide university health system during COVID-19. Telemed J E Health. Aug 2023;29(8):1134-1142. [doi: ] [Medline: 36576982]

44. Cockrell HC, Maine RG, Hansen EE, et al. Environmental impact of telehealth use for pediatric surgery. J Pediatr Surg. Dec 2022;57(12):865-869. [doi: ] [Medline: 35918239]

45. Gunn AH, Murray EM, Patel MR, Mentz RJ. Carbon emissions and air pollution savings among telehealth visits for cardiology appointments. Am Heart J Plus. Sep 2024;45:100435. [doi: ] [Medline: 39211004]

46. Jiang CY, Strohbehn GW, Dedinsky RM, et al. Teleoncology for veterans: high patient satisfaction coupled with positive financial and environmental impacts. JCO Oncol Pract. Sep 2021;17(9):e1362-e1374. [doi: ] [Medline: 34406817]

47. Sillcox R, Blaustein M, Khandelwal S, Bryant MK, Zhu J, Chen JY. Telemedicine use decreases the carbon footprint of the bariatric surgery preoperative evaluation. Obes Surg. Aug 2023;33(8):2527-2532. [doi: ] [Medline: 37407773]

48. Penaskovic KM, Zeng X, Burgin S, Sowa NA. Telehealth: reducing patients’ greenhouse gas emissions at one academic psychiatry department. Acad Psychiatry. Oct 2022;46(5):569-573. [doi: ] [Medline: 35997996]

49. Cummins MR, Shishupal S, Wong B, et al. Travel distance between participants in US telemedicine sessions with estimates of emissions savings: observational study. J Med Internet Res. May 15, 2024;26:e53437. [doi: ] [Medline: 38536065]

50. Patel KB, Gonzalez BD, Turner K, et al. Estimated carbon emissions savings with shifts from in-person visits to telemedicine for patients with cancer. JAMA Netw Open. Jan 3, 2023;6(1):e2253788. [doi: ] [Medline: 36719682]

51. Grabski DF, Meyer MJ, Gander JW. Pediatric telemedicine visits reduce greenhouse gas emissions. J Clim Chang Health. 2024;17:100309. [doi: ] [Medline: 41647654]

52. Moore L, Balmer F, Woywodt A. The environmental impact of changing to virtual renal transplant aftercare: 2-year experience with a single outpatient clinic. Future Healthc J. Mar 2024;11(1):100004. [doi: ] [Medline: 38646053]

53. Miah S, Dunford C, Edison M, et al. A prospective clinical, cost and environmental analysis of a clinician-led virtual urology clinic. Ann R Coll Surg Engl. Jan 2019;101(1):30-34. [doi: ] [Medline: 30286648]

54. King J, Poo SX, El-Sayed A, et al. Towards NHS zero: greener gastroenterology and the impact of virtual clinics on carbon emissions and patient outcomes. A multisite, observational, cross-sectional study. Frontline Gastroenterol. 2023;14(4):287-294. [doi: ] [Medline: 37409339]

55. Bhavanasi BA, Kulkarni S. Impact of virtual clinics on streamlining NHS outpatient waiting lists and carbon emissions. Foot Ankle Orthop. Oct 2024;9(4):24730114241305603. [doi: ] [Medline: 39698375]

56. Dorrian C, Ferguson J, Ah-See K, et al. Head and neck cancer assessment by flexible endoscopy and telemedicine. J Telemed Telecare. 2009;15(3):118-121. [doi: ] [Medline: 19364891]

57. Lewis D, Tranter G, Axford AT. Use of videoconferencing in Wales to reduce carbon dioxide emissions, travel costs and time. J Telemed Telecare. 2009;15(3):137-138. [doi: ] [Medline: 19364897]

58. Connor MJ, Miah S, Edison MA, et al. Clinical, fiscal and environmental benefits of a specialist-led virtual ureteric colic clinic: a prospective study. BJU Int. Dec 2019;124(6):1034-1039. [doi: ] [Medline: 31206221]

59. Sellars H, Ramsay G, Sunny A, Gunner CK, Oliphant R, Watson AJM. Video consultation for new colorectal patients. Colorectal Dis. Sep 2020;22(9):1015-1021. [doi: ] [Medline: 32628286]

60. Tarpani RRZ, Gallego-Schmid A. Environmental impacts of a digital health and well-being service in elderly living schemes. Clean Environ Syst. Mar 2024;12:100161. [doi: ]

61. Bartlett S, Keir S. Calculating the carbon footprint of a geriatric medicine clinic before and after COVID-19. Age Ageing. Feb 2, 2022;51(2):afab275. [doi: ] [Medline: 35134839]

62. Blenkinsop S, Foley A, Schneider N, Willis J, Fowler HJ, Sisodiya SM. Carbon emission savings and short-term health care impacts from telemedicine: an evaluation in epilepsy. Epilepsia. Nov 2021;62(11):2732-2740. [doi: ] [Medline: 34462915]

63. Lathan R, Hitchman L, Walshaw J, et al. Telemedicine for sustainable postoperative follow-up: a prospective pilot study evaluating the hybrid life-cycle assessment approach to carbon footprint analysis. Front Surg. 2024;11:1300625. [doi: ] [Medline: 38562585]

64. Masino C, Rubinstein E, Lem L, Purdy B, Rossos PG. The impact of telemedicine on greenhouse gas emissions at an academic health science center in Canada. Telemed J E Health. Nov 2010;16(9):973-976. [doi: ] [Medline: 20958198]

65. Mainer-Pearson G, Stewart K, Williams K, et al. Estimating patient and family costs and CO2 emissions for telehealth and in-person health care appointments in British Columbia, Canada: geospatial mixed methods study. J Med Internet Res. Feb 19, 2025;27:e56766. [doi: ] [Medline: 39969971]

66. Galts C, Anvari S, Kim A, Leontiadis G, Armstrong D. Sustainable practice in gastroenterology: travel-related CO_2_ emissions for gastroenterology clinic appointments in Canada. J Can Assoc Gastroenterol. Feb 2025;8(1):7-12. [doi: ] [Medline: 39906278]

67. Heffernan A, Lalande A, Chadha R, MacNeill A, Chadha NK. Carbon savings potential of virtual care in obstructive sleep apnea and otitis media with effusion. Laryngoscope Investig Otolaryngol. Apr 2024;9(2):e1221. [doi: ] [Medline: 38623079]

68. Lambert P, Musto G, Thiessen M, Czaykowski P, Decker K. Impact of cancer-related virtual visits on travel distance, travel time, and carbon dioxide (CO_2_) emissions during the COVID-19 pandemic in Manitoba, Canada. Curr Oncol. Jun 21, 2023;30(7):5973-5983. [doi: ] [Medline: 37504307]

69. Smith AC, Kimble RM, O’brien A, Mill J, Wootton R. A telepaediatric burns service and the potential travel savings for families living in regional Australia. J Telemed Telecare. Dec 2007;13(3_suppl):76-79. [doi: ]

70. Barakat-Johnson M, Kita B, Jones A, et al. The viability and acceptability of a virtual wound care command centre in Australia. Int Wound J. Nov 2022;19(7):1769-1785. [doi: ] [Medline: 35607997]

71. Schulz TR, Kanhutu K, Sasadeusz J, Watkinson S, Biggs BA. Using telehealth to improve access to hepatitis C treatment in the direct-acting antiviral therapy era. J Telemed Telecare. Apr 2020;26(3):180-185. [doi: ] [Medline: 30336724]

72. Andrew N, Barraclough KA, Long K, et al. Telehealth model of care for routine follow up of renal transplant recipients in a tertiary centre: a case study. J Telemed Telecare. May 2020;26(4):232-238. [doi: ] [Medline: 30449243]

73. Savoldelli A, Landi D, Rizzi C. Exploring the environmental impact of telemedicine: a life cycle assessment. Stud Health Technol Inform. Apr 26, 2024;313:81-86. [doi: ] [Medline: 38682509]

74. Materazzo M, Facchini A, Garozzo D, et al. Maintaining good practice in breast cancer management and reducing the carbon footprint of care: study protocol and preliminary results. World Cancer Research J. 2022;9:1-7. [doi: ]

75. Papavero SC, Fracasso A, Ramaglia P, Cicchetti A, de Belvis AG, Ferrara FM. Telemedicine has a social impact: an Italian national study for the evaluation of the cost-opportunity for patients and caregivers and the measurement of carbon emission savings. Telemed J E Health. Aug 2023;29(8):1252-1260. [doi: ] [Medline: 36637858]

76. Desenzi Ciaralo PP, Guerreiro Cardoso PF, Minamoto H, Bibas BJ, Ribeiro de Carvalho CR, Pego-Fernandes PM. Estimated carbon emissions and support cost savings to telemedicine for patients with tracheal diseases. Int J Med Inform. Mar 2025;195:105757. [doi: ] [Medline: 39671853]

77. Prosperi Desenzi Ciaralo P, Guerreiro Cardoso PF, Minamoto H, Bibas BJ, Ribeiro de Carvalho CR, Pego-Fernandes PM. Implementation and results of a dedicated telemedicine program (TeleTrachea) for patients with tracheal diseases. Telemed J E Health. May 2024;30(5):1317-1324. [doi: ] [Medline: 38109228]

78. Rani PK, Khanna RC, Ravindrane R, et al. Teleophthalmology at a primary and tertiary eye care network from India: environmental and economic impact. Eye (Lond). Aug 2024;38(11):2203-2208. [doi: ] [Medline: 38253864]

79. Kwon C, Essayei L, Spencer M, et al. The environmental impacts of electronic medical records versus paper records at a large eye hospital in India: life cycle assessment study. J Med Internet Res. Feb 6, 2024;26:e42140. [doi: ] [Medline: 38319701]

80. Vidal-Alaball J, Franch-Parella J, Lopez Seguí F, Garcia Cuyàs F, Mendioroz Peña J. Impact of a telemedicine program on the reduction in the emission of atmospheric pollutants and journeys by road. Int J Environ Res Public Health. Nov 8, 2019;16(22):4366. [doi: ] [Medline: 31717386]

81. Maria MS, Silvia AN, Beatriz DG, Andrew D, Guillermo PF. Health care in rural areas: proposal of a new telemedicine program assisted from the reference health centers, for a sustainable digitization and its contribution to the carbon footprint reduction. Heliyon. Jul 2022;8(7):e09812. [doi: ] [Medline: 35800252]

82. Muschol J, Heinrich M, Heiss C, et al. Economic and environmental impact of digital health app video consultations in follow-up care for patients in orthopedic and trauma surgery in Germany: randomized controlled trial. J Med Internet Res. Nov 24, 2022;24(11):e42839. [doi: ] [Medline: 36333935]

83. Arndt EM, Jansen TR, Bojko J, et al. COVID-19 measures as an opportunity to reduce the environmental footprint in orthopaedic and trauma surgery. Front Surg. 2023;10:959639. [doi: ] [Medline: 37123547]

84. Filfilan A, Anract J, Chartier-Kastler E, et al. Positive environmental impact of remote teleconsultation in urology during the COVID-19 pandemic in a highly populated area. Prog Urol. Dec 2021;31(16):1133-1138. [doi: ] [Medline: 34454847]

85. Oppelaar MC, Bannier M, Reijers MHE, et al. Remote monitoring and teleconsultations can reduce greenhouse gas emissions while maintaining quality of care in cystic fibrosis. Front Digit Health. 2024;6:1469860. [doi: ] [Medline: 39512858]

86. Li Z, Xiang L, Ning J, Li W, Huang Y, Xiao X. Pathways to sustainable health care development: study on the carbon reduction potential of telemedicine in China. J Med Internet Res. 2025;27:e63927. [doi: ]

87. Özen F, Kaynar AH, Korkut AK, Teker Açıkel ME, Kaynar ZD, Kaynar AM. The role of telemedicine towards improved sustainability in healthcare and societal productivity in Turkey. PLoS One. 2024;19(12):e0314986. [doi: ] [Medline: 39637073]

88. Meryk A, Salvador C, Kropshofer G, et al. Pioneering sustainable treatment delivery in childhood leukemia through synchronous telemedicine-a pilot study. Int J Cancer. Mar 15, 2025;156(6):1247-1255. [doi: ] [Medline: 39520275]

89. Giacomo P, Håkansson P. A Method to Measure the Reduction of CO 2 Emissions in E-Health Applications, User Centred Networked Health Care. IOS Press; 2011:970-974. [doi: ]

90. Croghan SM, Rohan P, Considine S, et al. Time, cost and carbon-efficiency: a silver lining of COVID era virtual urology clinics? Ann R Coll Surg Engl. Sep 2021;103(8):599-603. [doi: ] [Medline: 34464571]

91. Schmitz-Grosz K, Sommer-Meyer C, Berninger P, et al. A telemedicine center reduces the comprehensive carbon footprint in primary care: a monocenter, retrospective study. J Prim Care Community Health. 2023;14:21501319231215020. [doi: ] [Medline: 38041467]

92. Dieperink KB, Vestergaard LV, Møller PK, Tolstrup LK. Using video consultations for clinical assessment and decision of treatment readiness before chemotherapy: a mixed-methods study among patients with gastrointestinal cancer and oncology nurses. Digit Health. 2023;9:20552076231197415. [doi: ] [Medline: 37675059]

93. Oliveira TC, Barlow J, Gonçalves L, Bayer S. Teleconsultations reduce greenhouse gas emissions. J Health Serv Res Policy. Oct 2013;18(4):209-214. [doi: ] [Medline: 23945677]

94. Holmner A, Ebi KL, Lazuardi L, Nilsson M. Carbon footprint of telemedicine solutions-unexplored opportunity for reducing carbon emissions in the health sector. PLoS One. 2014;9(9):e105040. [doi: ] [Medline: 25188322]
