# Supplementary material for: Environmental Impact of Digital Health Care Technologies in Reducing Carbon Footprints: Scoping Review
Source: J Med Internet Res. 2026 Oct 21;28:e88341. doi: 10.2196/88341 (PMC13717332; doi:10.2196/88341)
Supplement: Supplementary file 5 [file jmir-v28-e88341-s005.docx]

**Software programs and conversion factors or emissions calculators used in environmental impact assessment**

| **Software programs and conversion factors/emissions calculators used in environmental impact assessment** | **References** |
| --- | --- |
| Travel distance | |
| Google maps | Marković et al [12]  Kellerman et al [28]  Roy et al [33]  Dacones et al [36]  Thota et al [38]  Iaccarino et al [40]  Jiang et al [46]  Miah et al [53]  King et al [54]  Bhavanasi et al [55]  Connor et al [58]  Masino et al [64]  Galts et al [66]  Smith et al [69]  Barakat‐Johnson et al [70]  Papavero et al [75]  Ciaralo et al [76]  Vidal-Alaball et al [80]  Filfilan et al [84]  Oppelaar et al [85]  Özen et al [87]  Meryk et al [88] |
| Mapping software | Danesh et al [27] |
| Python (version 3.7; Python Software Foundation) with Baidu Maps | Li et al [86] |
| Python 3.6 script utilising Google Maps software | Want et al [35] |
| penStreetMap package (version 4.0) and calculating with R (version 4.2; R Core Team) | Mainer-Pearson [65] |
| Road distance - a standard route planner (Automobile Association Limited) | Dorrian et al [56] |
| Great circle distance between airports - Vincenty’s formula | Dorrian et al [56] |
| MapPoint 2013 (Microsoft Corporation, Redmond, Wash) to geocode patient and telemedicine client site addresses | Sellars et al [59] |
| AA Route Planner© and Google Maps© | Croghan et al [90] |
| Geo-Coded National Address File (GNAF) | Andrew et al [72] |
| ArcGIS tools | Whetten and Yonas [37] |
| ArcGIS online and Google Maps rooting tools | Blenkinsop et al [62] |
| Geo-mapping database13 | Moore et al [42] |
| Geocoding with Nominatim software | Schmitz-Grosz et al [91] |
| Haversine formula in the geosphere software | Cockrell et al [44] |
| ArcGIS Pro software (version 3.2). PM2.5, CO | Gunn et all [45] |
| ArcGIS Pro version 2.7 to geocode | Penaskovic et al [48] |
| Google Distance Matrix Application Programming Interface | Cummins et al [49] |
| R package gmapsdistance | Lambert et al [68] |
| CF and GHGs emission assessment: Travel | |
| UK government conversion factors | Moore et al [52]  Lewis et al [57]  Oppelaar [85] |
| Carbon emissions calculator provided by Carbon footprint | Miah et al [53]  Croghan [90] |
| Conversion factors used by international organisations to report on 2020 GHG | Filfilan et al [84] |
| GHG and pollution releases using emission coefficients provided to Metrolinx by Transport Canada | Masino et al [64] |
| Conversion factors available in the Department for Business, Energy and Industrial Strategy | King et al [54] |
| Carbon footprint of the average UK petrol car emits 0.28kgCO2e per mile | Bhavanasi and Kulkarni [55] |
| US Environmental Protection Agency ratios | Danesh et al [27]  Kellerman et al [28]  Wong et al [30]  Mojdehbakhsh et al [32]  Robinson et al [34]  Whetten et al [37]  Thota et al [38]  Iaccarino et al [40]  Moore et al [42]  Gunn et al [45]  Sillcox et al [47]  Penaskovic et al [48]  Cummins et al [49]  Patel et al [50]  Grabski et al [51]  Andrew et al [72]  Dieperink et al [92] |
| Greenhouse Gas Protocol (GHG Protocol) adapted to the Brazilian reality (Programa Brasileiro GHG Protocol) | Ciaralo et al [76] |
| Corresponds to a gasoline vehicle with an average consumption of 7.4 L/100 km in European countries | Meryk et al [88] |
| Standardised emission factor according to Environmental Protection Agency (EPA) data for Canada | Galts et al [66] |
| Methodology of the Department for Environment, Food and Rural Affairs | Dorrian et al [56] |
| A calculator supplied by Carbon Footprint by Kmietowicz (2016) | Connor et al [58] |
| Carbon footprint using the internet-based tool Map My Emissions (https://www.mapmyemissions.com/home) | Sellars et al [59] |
| Carbon footprint associated with air travel by a tool provided by a United Nations agency, the International Civil Aviation Organisation. (https://www.icao.int/environmental-protection/Carbonoffset/Pages/default.aspx) | Sellars et al [59] |
| GHG emissions for travel: based on the data from the Federal Highway Administration (FHWA) and the United States Environmental Protection Agency (US EPA) | Roy et al [33] |
| CO_2_ emissions using the IDESAM emissions calculator | Ciaralo et al [77] |
| Carbon emission values specific to India | Rani et al [78] |
| The Australian average for passenger cars is 258 g of carbon produced per kilometre | Schulz et al [71] |
| GHG emissions by data from the Department’s health management report and the emission factors of the vehicles | Maria et al [81] |
| GHG emissions by tool for the Reduction and Assessment of Chemical and other environmental Impacts (TRACI) 2.1 Life Cycle Impact Assessment (Characterisation) Methodology and United States 2008 Normalisation Set | Dacones et al [36] |
| Automobile CO emission of 411 g CO per mile, converting to metric tonnage | Dacones et al [36] |
| Department for Environment, Food and Rural Affairs (DEFRA) published values for petrol emissions per mile | Bartlett and Keir [61] |
| Conversion factors for travel published by the UK Department for Business, Energy and Industrial Strategy (BEIS) | Blenkinsop et al [62] |
| Regulation (EU) 2019/631 of the European Parliament and of the Council of 17 April 2019 Setting CO_2_ Emission Performance Standards for New Passenger Cars and for New Light Commercial Vehicles, and Repealing Regulations (EC) No 443/2009 and (EU) No 510/2011; 2019 | Papavero et al [75] |
| Emission factors from Mobitool, a Swiss platform for mobility management tools and environmental data processing and CO_2_-Emissionen pro Kilowattstunde Strom steigen 2021 Wieder an. Umweltbundesamt | Schmitz-Grosz et al [91] |
| U.S. Environmental Protection Agency (EPA) and the Bureau of Transportation’s estimated U.S. average vehicle emission rates in grams per mile per vehicle for 2021 | Sharma et al [43] |
| GHG analysis: by 2012 Guidelines to Defra/DECCs GHG Conversion Factors for Company Reporting to estimate direct and indirect GHG emissions | Oliveira et al [93] |
| Canada's National GHG Inventory Report with a corresponding EF of 2.462 kgCO_2_e/L/L.22 | Heffernan et al [67] |
| German Federal Environment Agency, published in the Handbook of Emission Factors (HBEFA) | Arndt et al [83] |
| Estimated metric tons of CO2 emissions: 206 g of CO_2_ per kilometre, which is an average based on newly registered vehicles in 2017 in Canada | Lambert et al [68] |
| The carbon emission data per km derived from the Global Change Data Lab | Özen et al [87] |
| Carbon Footprint Calculator. URL: <https://www.carbonfootprint.com/calculator.aspx> | Mainer-Pearson et al [65] |
| Carbon emissions and carbon footprint reductions based on tools such as CarbonFootprint.com and CO_2_Meter.com | Marković et al [12] |
| Emission by published data | Muschol et al [82] |
| 2008 Average Annual Emissions and Fuel Consumption for Gasoline-Fueled Passenger Cars and Light Trucks report, produced by the U.S. Environmental Protection Agency Office of Transportation and Air Quality | Dullet et al [31] |
| Emission of pollutants per km based on Mobility and emissions. Extra-urban cycle (gasoline and diesel fuel). Available at: <http://mobilitat.gencat.cat/ca/detalls/Article/mobilitat_emissions> | Vidal-Alaball et al[80] |
| Energy consumption | |
| French national conversion factor | Filfilan et al [84] |
| Emission factors for Ontario from Environment Canada’s National Inventory Report: Greenhouse Gas Sources and Sinks in Canada, 1990–2006 | Masino et al [64] |
| Annual household electricity consumption values published by the UK Household Electricity Survey | Bartlett et al [61] |
| Energy consumption published by the UK Department for Business, Energy and Industrial Strategy (BEIS) | Blenkinsop et al [62] |
| British Columbia electrical grid intensity of 0.097 kgCO2e/kWh to determine carbon emissions associated with virtual visits, assuming a 30-minute appointment | Heffernan et al [67] |
| Personal protective equipment | |
| Values published for PPE use in the UK by Rizan et al. for Life cycle assessment | Bartlett et al [61] |
| Domestic wastes, wastewater and clean water usage | |
| Data published by DEFRA | Bartlett et al [61] |
| Emissions from telecommunication | |
| According to Ong et al | Bartlett et al [61] |
| Smartphone emission | |
| Using the published value of 63 kg of CO2/year of emissions if using a smartphone for 1 hr per day, based on Berners-Lee 2020 | Penaskovic et al [48] |
| Electricity | |
| Based on fixed-line energy transmission estimates using the US Environmental Protection Agency conversion rate of 4.33 | Cummins et al [49] |
| Infrastructure | |
| Based on available conversion factors | King et al [54] |
| Emission equivalencies | |
| Standard agricultural method, which assumes planting five trees to ensure one reaches maturity (Trees for the Future; [www.trees.org](http://www.trees.org)) | Miah et al [53]  Connor et al [58] |
| Estimation of the number of trees needed to offset the carbon footprint based on Forests, health and climate change | Oppelaar et al [85] |
| 21.77 kg CO2 per year for a mature tree by Komninos 2022 | Galts et al [66] |
| Tree Council of Ireland's online information page | Croghan et al [90] |
| One mature tree offsets on average 0.0029 t carbon dioxide per year (<https://trees.org/carboncalculator> | Barakat‐Johnson et al [70] |
| The estimated number of trees required to absorb the emissions by Holmner et al | Iaccarino et al [40] |
| Carbon sequestration equivalent-land in acres by the US Environmental Protection Agency | Turley et al [26] |
| Number of barrels of gasoline burned based on the US Environmental Protection Agency | Cockrell et al [44] |
| Electricity required for a family of four’s home using Holmner et al | Iaccarino et al [40] |
| Life cycle assessment | |
| - Production and end-of-life disposal: Computer device manufacturer’s website - Products that did not have an available LCA: Known economic information to estimate emissions for . - Travel distances: Holmner et al. and the Ecoinvent database | Martinez et al [29] |
| - Energy consumption per unit mileage of the car throughout its life cycle: Ecoinvent (version 3.9; Ecoinvent Association) - The energy consumption intensity of the software and health services: conversion of CO2e emissions per unit of standard carbon for | Li et al [86] |
| - Mobile and fixed broadband networks: based on published data on | Di Giacomo and Håkansson [89] |
| - Entire life cycle: by means of the FootprintCalc database | Savoldelli et al [73] |
| - The ReCiPe 2016(H) V1.06 | Tarpani and Gallego-Schmid [60] |
| - CO_2_e emissions associated with PEC visits and laboratory and radiographic testing: OpenLCA v1.8 interface software - Emissions due to vehicular commuting: the U.S. EPA emissions calculator and Google Maps software | Wang et al [35] |
| - LCA: Ecoinvent database. - When a specific product’s LCA was not available, an economic input–output LCA utilising known economic information to estimate associated emissions | Sillcox et al [39] |
| - Life cycle emissions of clinic visits: LCA software SimaPro 9.3.0.240 and LCI database Ecoinvent v3.82 - The impact assessment using the US Environmental Protection Agency’s TRACI 2.1 v1.06/ US 2008 (Tool for Reduction and Assessment of Chemicals and Other Environmental Impact | Thiel et al [41] |
| - Anaesthetic gases: emissions factors provided by the Sustainable Development Unit and Association of Anaesthetists Anaesthetic Gas Calculator, Emissions factors for surgical intervention. - Electricity: the Department for Environment, Food, and Rural Affairs (DEFRA) and Business, Energy, and Industrial Strategy (BEIS) GHG conversion factors. - Water use: DEFRA/BEIS GHG conversion factors. - Individual clinic item costings: NHS supply chain online catalogue - Radiological investigation costs: The National Institute for Health Research (NIHR) interactive costing tool for investigation and intervention tariff - Medication costs: accompanying emissions factors for pharmaceutical data. For oral medications, empty blister packs were weighed and quantified before mapping incineration factors. - Hourly cost data for hospital clinicians, general practitioners, and community nurses: National tariff data from the Personal Social Services Research Unit (PSSRU). - Patient travel emissions: emissions factor for method of transport (DEFRA/BEIS conversion factors) | Lathan et al [63] |
| - Life cycle inventory: SimaPro (version 9.3.0.2; PréConsultants) and the Ecoinvent (version 3.8; Ecoinvent) database | Kwon et al [79] |
| - LCA: the strategy described in Ong et al. - Travelling: based on the research by Lenzen et al. According to Lenzen, the total carbon cost of a car is 0.86 kgCO2e/km | Holmner et al [94] |
